# The CTBP2-PCIF1 complex regulates m^6^Am modification of mRNA in head and neck squamous cell carcinoma

**DOI:** 10.1172/JCI170173

**Published:** 2023-08-29

**Authors:** Kang Li, Jie Chen, Caihua Zhang, Maosheng Cheng, Shuang Chen, Wei Song, Chunlong Yang, Rongsong Ling, Zhi Chen, Xiaochen Wang, Gan Xiong, Jieyi Ma, Yan Zhu, Quan Yuan, Qi Liu, Liang Peng, Qianming Chen, Demeng Chen

**Affiliations:** 1 Otorhinolaryngology Hospital, The First Affiliated Hospital, Sun Yat-sen University, Guangzhou, China.; 2 Hospital of Stomatology, Guangdong Provincial Key Laboratory of Stomatology, Guanghua School of Stomatology, Sun Yat-sen University, Guangzhou, China.; 3 State Key Laboratory of Oral Diseases, National Clinical Research Center for Oral Diseases, West China Hospital of Stomatology, Sichuan University, Chengdu, China.; 4 Institute for Advanced Study, Shenzhen University, Shenzhen, China.; 5 Rice Research Institute, Guangdong Academy of Agricultural Sciences, Key Laboratory of Genetics and Breeding of High Quality Rice in Southern China (Co-construction by Ministry and Province), Guangzhou, China.; 6 Senior Department of Oncology, The Fifth Medical Center of PLA General Hospital, Fengtai District, Beijing, China.; 7 Stomatology Hospital, School of Stomatology, Zhejiang University School of Medicine, Zhejiang Provincial Clinical Research Center for Oral Diseases, Key Laboratory of Oral Biomedical Research of Zhejiang Province, Cancer Center of Zhejiang University, Engineering Research Center of Oral Biomaterials and Devices of Zhejiang Province, Hangzhou, China.

**Keywords:** Cell biology, Oncology, Epigenetics, Head and neck cancer

## Abstract

PCIF1 can mediate the methylation of *N*^6^,2′-*O*-dimethyladenosine (m^6^Am) in mRNA. Yet, the detailed interplay between PCIF1 and the potential cofactors and its pathological significance remain elusive. Here, we demonstrated that PCIF1-mediated cap mRNA m^6^Am modification promoted head and neck squamous cell carcinoma progression both in vitro and in vivo. CTBP2 was identified as a cofactor of PCIF1 to catalyze m^6^Am deposition on mRNA. CLIP-Seq data demonstrated that CTBP2 bound to similar mRNAs as compared with PCIF1. We then used the m^6^Am-Seq method to profile the mRNA m^6^Am site at single-base resolution and found that mRNA of *TET2*, a well-known tumor suppressor, was a major target substrate of the PCIF1-CTBP2 complex. Mechanistically, knockout of CTBP2 reduced PCIF1 occupancy on *TET2* mRNA, and the PCIF1-CTBP2 complex negatively regulated the translation of *TET2* mRNA. Collectively, our study demonstrates the oncogenic function of the epitranscriptome regulator PCIF1-CTBP2 complex, highlighting the importance of the m^6^Am modification in tumor progression.

## Graphical abstract

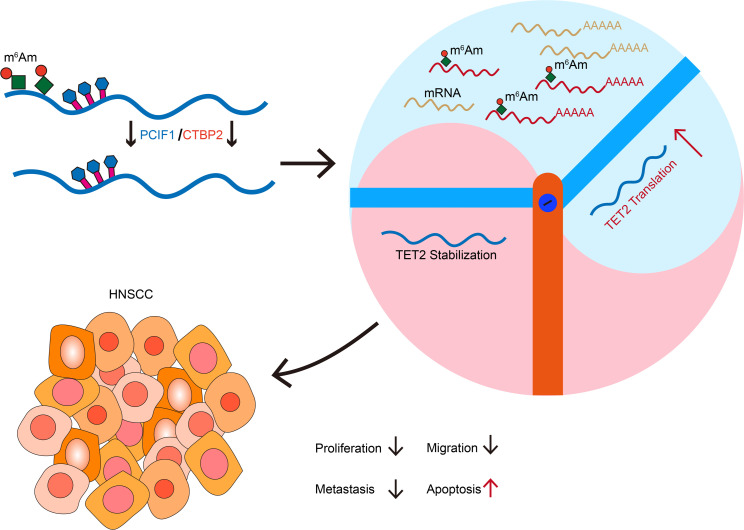

## Introduction

Recently, it has been increasingly evident that dysregulation of mRNA modifications and dysregulation of the modifiers are important factors in the aggressiveness and malignancy of tumors (1–4). Methylated modifications constitute more than 60% of all RNA modifications (5). To date, several kinds of eukaryotic mRNA methylated modifications have been characterized, including internal *N*^6^-methyladenosine (m^6^A) and 5-methylcytosine (5mC), *N*^6^-methyl-2′-*O*-methyladenosine (m^6^Am) within the cap and the internal regions, and *N*^7^-methylguanosine (m^7^G) at the cap (6). Compared with m^6^A modification, which is the most abundant mRNA modification, m^6^Am is highly prevalent and is found at the first encoded nucleotide position adjacent to the m^7^G cap in 30%–40% of mRNAs, more than those with m^6^A modification (7, 8). Reversible and dynamic changes of mRNA m^6^Am play important roles in tumorigenesis through interfering with the stability, decapping, and translation of mRNA targets, including a variety of key oncogenes and tumor suppressors (9).

The modification of m^6^Am is controlled by opposing activities of specific methyltransferases and demethylases. Phosphorylated RNA polymerase II CTD–interacting factor 1 (PCIF1) is the sole known methyltransferase to catalyze the m^6^Am marks present in the m^7^G cap–proximal nucleotide (10–13). However, it remains unknown whether modification of m^6^Am requires recruitment of cofactors to the target mRNA. On the other hand, fat mass– and obesity-associated protein (FTO) has been shown to preferentially demethylate cap m^6^Am of RNA transcripts (8). While the role of m^6^A modification in cancer biology is well known, the role of m^6^Am in tumor progression is still emerging (1, 14). Especially, newly developed methodologies have enabled the precise pinpointing of m^6^Am modifications across the transcriptome and the clarification of their functional outcomes. For example, the m^6^Am-Seq method developed by Sun and colleagues has successfully identified m^6^Am at single-base resolution based on a demethylation step that selectively removes m^6^Am while keeping m^6^A intact, allowing them to distinguish m^6^Am from m^6^A peaks (15).

In this study, we found that PCIF1 was overexpressed in head and neck squamous cell carcinoma (HNSCC) cancer tissues and served as a prognostic marker for HNSCC. Our loss-of-function and gain-of-function assays showed that PCIF1 was essential for tumorigenic properties of HNSCC. Moreover, PCIF1 interacted with C-terminal binding protein 2 (CTBP2) to deposit the m^6^Am in the m^7^G cap–proximal nucleotide. CTBP2 exerted m^6^Am modification by binding to PCIF1 in the nucleus to form the PCIF1-CTBP2 complex. Moreover, we identified Tet methylcytosine dioxygenase 2 (TET2) as a functional downstream target of PCIF1-CTBP2 complex in HNSCC. The m^6^Am modification on *TET2* mediated by PCIF1-CTBP2 negatively regulated the translation of *TET2* transcript. In addition, our mouse models indicated that PCIF1-CTBP2 complex was important for HNSCC development and progression. In summary, our results support a critical role of PCIF1-CTBP2 and m^6^Am modification in HNSCC development.

## Results

### Expression of PCIF1 and its association with clinicopathological parameters of HNSCC.

**Figure 1:**
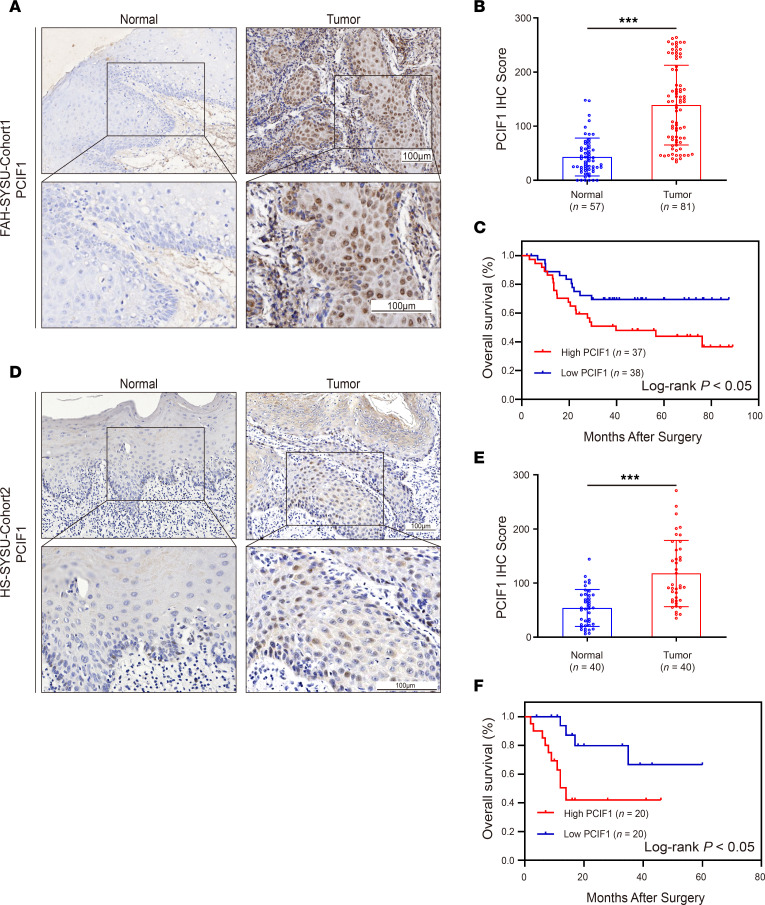
PCIF1 is upregulated in HNSCC patients and correlates with poor prognosis. (**A**) Representative images of PCIF1 staining in tumor and non-tumorous tissues from HNSCC patients (FAH-SYSU-Cohort1). Scale bars: 100 μm. (**B**) Quantification of PCIF1 staining score between tumor tissue samples (*n* = 81) and non-tumorous tissue samples (*n* = 57) from HNSCC patients (FAH-SYSU-Cohort1). ***P < 0.001 by 2-tailed unpaired Student’s *t* test. (**C**) Kaplan-Meier curve depicting the overall survival of patients with HNSCC (FAH-SYSU-Cohort1) stratified by PCIF1 expression levels. *P* values were calculated by log-rank test. (**D**) Representative images of PCIF1 staining in tumor and non-tumorous tissues from HNSCC patients (HS-SYSU-Cohort2). Scale bars: 100 μm. (**E**) Quantification of PCIF1 staining score between tumor tissue samples (*n* = 40) and non-tumorous tissue samples (*n* = 40) from HNSCC patients (HS-SYSU-Cohort2). ***P < 0.001 by 2-tailed unpaired Student’s *t* test. (**F**) Kaplan-Meier curve depicting the overall survival of patients with HNSCC (HS-SYSU-Cohort2) stratified by PCIF1 expression levels. *P* values were calculated by log-rank test.

To assess the mRNA expression level of *PCIF1* in HNSCC, we first analyzed The Cancer Genome Atlas (TCGA) Head-Neck Squamous Cell Carcinoma data sets. We found that mRNA expression of *PCIF1* was significantly higher in HNSCC patients than in non-cancer subjects (Supplemental Figure 1A; supplemental material available online with this article; https://doi.org/10.1172/JCI170173DS1). In addition, samples from a total of 121 patients with HNSCC from 2 independent local cohorts were used in this study. Cohort 1 comprised 81 HNSCCs obtained from The First Affiliated Hospital of Sun Yat-sen University (FAH-SYSU). The demographic, pathological, and clinical information of this cohort is provided in Supplemental Table 1. Immunohistochemical (IHC) staining of PCIF1 was performed to determine the protein expression of PCIF1 and its association with various clinicopathological features in 81 HNSCC tissues and 57 adjacent normal tissues. IHC results demonstrated that PCIF1 expression was mainly localized in the nucleus of tumor cells (Figure 1A) and that the expression of PCIF1 in HNSCC tissues was significantly higher than that in normal tissues (Figure 1B). There were no statistically significant correlations between PCIF1 expression and T classification (Supplemental Figure 1B). However, PCIF1 expression was significantly associated with tumor stage, tumor grade, and lymph node metastasis status in cohort 1 (Supplemental Figure 1, C–E). As shown by Kaplan-Meier log-rank analysis of cohort 1, higher PCIF1 levels correlated with poorer overall survival rates (Figure 1C). Cohort 2 included 40 HNSCC patients with tumors and matched normal adjacent tissues acquired from the Hospital of Stomatology, Sun Yat-sen University (HS-SYSU). Clinicopathological data for this cohort are listed in Supplemental Table 2. Similarly, PCIF1 was substantially higher in HNSCC than in matched normal tissues (Figure 1, D and E). Correlation of high expression of PCIF1 with the clinicopathological features in cohort 2 was also evaluated. Statistically significant correlations were observed between PCIF1-high character and T classification, tumor stage, tumor grade, and lymph node metastasis status (Supplemental Figure 1, F–I). Lastly, there was also a statistically significant difference in overall survival rates (*P* < 0.05) between patients with PCIF1-high and PCIF1-low tumors (Figure 1F).

### PCIF1 is essential for human HNSCC tumorigenesis.

**Figure 2:**
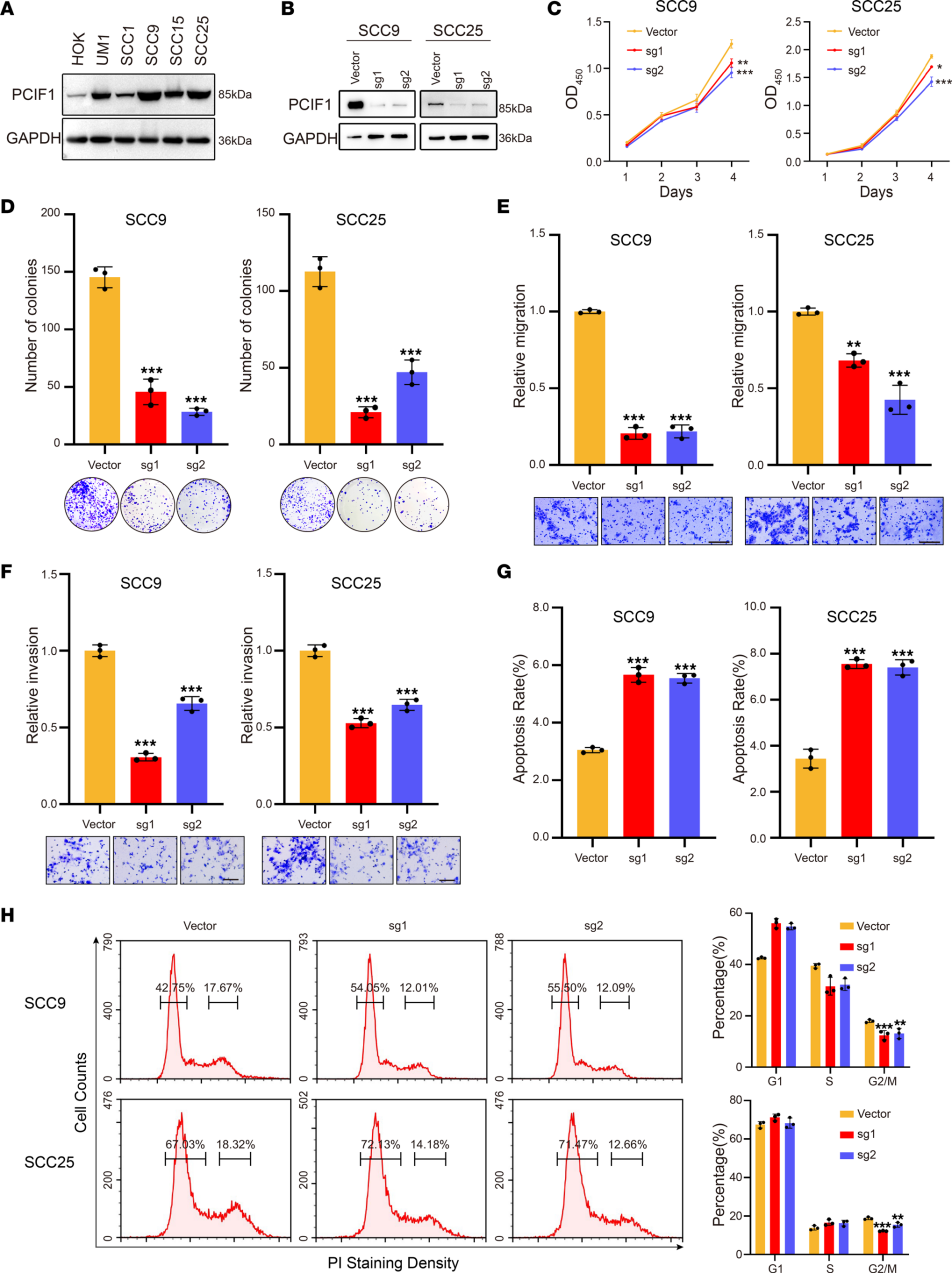
Knockout of PCIF1 suppresses the HNSCC malignant phenotype. (**A**) Western blotting analyses of the PCIF1 expression in the cell lines. (**B**) Western blotting analyses detecting the PCIF1 expression in SCC9 (left) and SCC25 (right) control cells and PCIF1-KO cells. (**C**) Cell Counting Kit-8 (CCK8) assay of cell viability in control and PCIF1-KO cells (*n* = 3). *P < 0.05, **P < 0.01, ***P < 0.001 by 1-way ANOVA, Dunnett’s test. (**D**) Colony formation assay detecting the colony ability of control and PCIF1-KO cells (*n* = 3). ***P < 0.001 by 1-way ANOVA with Tukey’s multiple-comparison test. (**E** and **F**) The cell migration (**E**) and invasion (**F**) ability of control and PCIF1-KO cells was determined by Transwell assay (*n* = 3). **P < 0.01, ***P < 0.001 by 1-way ANOVA with Tukey’s multiple-comparison test. Scale bar: 100 μm. (**G**) Flow cytometry assay for cell apoptosis in control and PCIF1-KO cells. Bottom: Representative images. Top: Quantification data (*n* = 3). ***P < 0.001 by 1-way ANOVA with Tukey’s multiple-comparison test. (**H**) Cell cycle progression was detected by flow cytometric analyses in control and PCIF1-KO cells (*n* = 3). **P < 0.01, ***P < 0.001 by 1-way ANOVA with Tukey’s multiple-comparison test.

To explore the role of PCIF1 in HNSCC, Western blotting was performed to examine the expression pattern of PCIF1 in HNSCC cell lines and normal human oral keratinocytes (HOKs). We found higher levels of PCIF1 in all 5 HNSCC cell lines compared with HOKs; SCC9 and SCC25 displayed the highest level of PCIF1 expression among all cell lines (Figure 2A). Subsequently, we generated SCC9 and SCC25 PCIF1-knockout (PCIF-KO) cells using the CRISPR/Cas9 system with 2 independent single-guide RNAs (sgRNAs). A marked decrease in PCIF1 protein level in both cell lines was observed by Western blot after CRISPR/Cas9 transfection (Figure 2B). We found there was significantly less cell growth by the PCIF1-KO cells than by the control cells (Figure 2C). In addition, depletion of PCIF1 resulted in reduced colony-forming ability of the SCC9 and SCC25 cells (Figure 2D). Next, we performed flow cytometric analysis to examine whether the cell cycle was altered. We found a decrease in the percentage of cells in G_2_/M phase, whereas the percentages of cells in G_1_ and S phases were not markedly affected (Figure 2H). We then carried out Transwell assay to study the effect of PCIF1 on HNSCC cell migration and invasion. Our results showed that PCIF1 KO led to a reduced number of migrating and invasive cells as compared with control cells (Figure 2, E and F). Furthermore, we found an increased percentage of apoptotic cells following PCIF1 KO in HNSCC cells (Figure 2G).

Complementarily, we overexpressed PCIF1 in SCC1 cells, which displayed the lowest expression level of PCIF1 compared with other HNSCC cell lines (Figure 2A and Supplemental Figure 2A). We found that exogenous expression of wild-type (WT) PCIF1 elicited increased levels of cell proliferation and colony-forming ability and percentage of cells in G_2_/M phase (Supplemental Figure 2, B–D). However, overexpression of mutant PCIF1 (N553A, a key residue for the m^6^Am methyltransferase activity of PCIF1) (1) failed to largely promote cell growth and proliferation (Supplemental Figure 2, A and D). In addition, overexpression of WT PCIF1 promoted the migratory and invasive abilities of HNSCC cells, whereas mutant PCIF1 was not able to do so (Supplemental Figure 2, E and F). Finally, WT PCIF1, but not mutant PCIF1, significantly reduced the percentage of SCC1 cells that were apoptotic (Supplemental Figure 2G). These data demonstrated that PCIF1 promoted tumorigenic properties of HNSCC cells dependent on its intact activity.

### PCIF1 interacts with CTBP2 to catalyze m^6^Am modification on mRNA.

**Figure 3:**
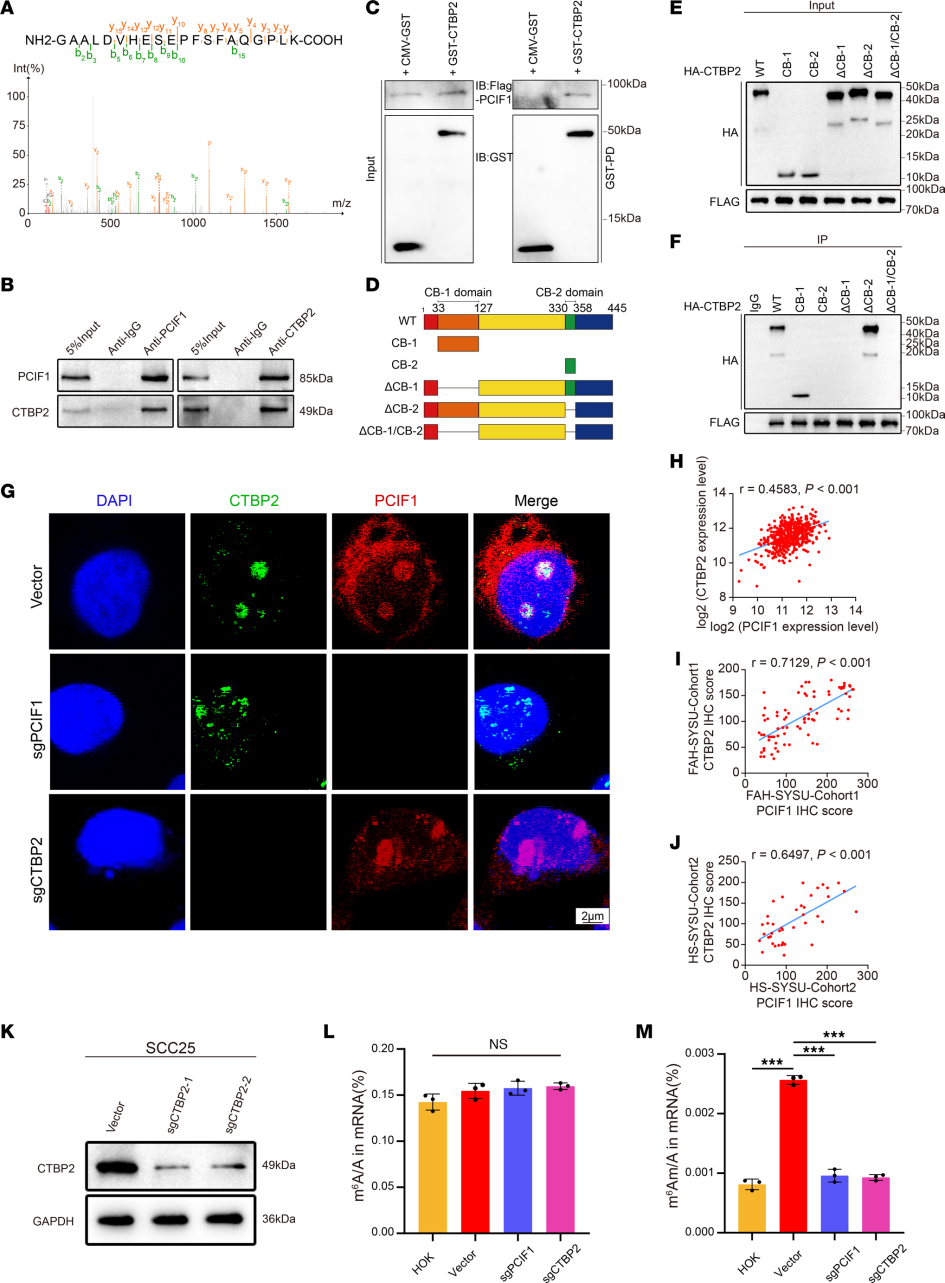
CTBP2 and PCIF1 interact and co-catalyze m^6^Am modification on mRNA. (**A**) The CTBP2 peptide identified in MS analysis after coimmunoprecipitation assay. (**B**) Immunoprecipitation and immunoblotting analysis of the interaction between CTBP2 and PCIF1. (**C**) GST pull-down assay of recombinant GST-CTBP2 and FLAG-PCIF1 proteins. The coprecipitated CTBP2 and PCIF1 proteins were detected by Western blot with anti-GST and anti-FLAG antibodies. (**D**) Strategy of CTBP2 variant proteins for mapping interaction domains with PCIF1. (**E** and **F**) Mapping of the binding domain of CTBP2 shows the potential binding site. The lysates were immunoprecipitated with anti-FLAG antibodies, followed by immunoblotting with anti-HA and anti-FLAG antibodies. Anti-IgG antibody was used as a negative control. (**G**) Analysis of colocalization of CTBP2 (green) with PCIF1 (red) by double immunofluorescence staining in control cells, PCIF1-KO cells, and CTBP2-KO cells. Nuclei are stained with DAPI (blue). Scale bar: 2 μm. (**H**–**J**) Pearson’s correlation analysis showed a positive correlation between CTBP2 and PCIF1 expression according to TCGA data (**H**, *n* = 520), FAH-SYSU-Cohort1 (**I**, *n* = 57), and HS-SYSU-Cohort2 (**J**, *n* = 40). *P* value was calculated by Pearson’s correlation coefficient test. (**K**) Western blotting analyses detecting the CTBP2 expression in SCC25 control cells and CTBP2-KO cells. (**L** and **M**) LC-MS/MS quantification of the m^6^A/A ratio (**L**) and m^6^Am/A ratio (**M**) in mRNA obtained from HOK cells, control cells, PCIF1-KO cells, and CTBP2-KO cells (*n* = 3). *P* > 0.05, ***P < 0.001 by 1-way ANOVA with Tukey’s multiple-comparison test.

In order to explore the multifunction and identify the unknown protein binding partners of PCIF1, we pulled down the endogenous PCIF1 proteins using specific antibody in SCC25 cells. Replicates of PCIF1 antibody pull-down samples and an IgG control sample were then used for mass spectrometry isobaric tags for relative and absolute quantitative (MS-iTRAQ) analysis. After subtracting background proteins in the IgG sample, we obtained a total of 103 common candidate proteins in both pull-down samples (Supplemental Table 3). Notably, PCIF1 was consistently pulled down in both samples, highlighting the quality of our MS-iTRAQ results. Importantly, CTBP2 showed the highest enrichment in both samples among all candidate proteins (Figure 3A and Supplemental Table 3), suggesting a possible interaction between PCIF1 and CTBP2 proteins. Besides, to confirm our results, we performed immunoprecipitation–Western blotting and found positive interaction between endogenous PCIF1 and CTBP2 proteins in SCC25 cells (Figure 3B). Moreover, glutathione-*S*-transferase (GST) pull-down assay using purified recombinant PCIF1 (FLAG-PCIF1) and CTBP2 (GST-CTBP2) proteins demonstrated a direct interaction between PCIF1 and CTBP2 (Figure 3C). To define the domains of CTBP2 that interacted with PCIF1, an in vitro mapping experiment was conducted. Hemagglutinin-tagged (HA-tagged) CTBP2 domains, including full-length CTBP2 (WT), CtBP-binding motif-1 (CB-1), CtBP-binding motif-2 (CB-2), CTBP2 without CtBP-binding motif-1 (ΔCB-1), CTBP2 without CtBP-binding motif-2 (ΔCB-2), and CTBP2 without CtBP-binding motif-1 or CtBP-binding motif-2 (ΔCB-1/CB-2), were coexpressed with PCIF1 in SCC25 cells. PCIF1 was detected in the CtBP-binding motif-1 precipitates but not in other conditions, indicating that CtBP-binding motif-1 was responsible for the interactions with PCIF1 (Figure 3, D–F). Next, our double immunofluorescent staining results revealed a strong colocalization of PCIF1 with CTBP2 in the nucleus of SCC25 cells using a confocal scanning laser microscope (Figure 3G). Additionally, correlation analysis revealed that *PCIF1* is highly correlated with *CTBP2* in TCGA data set (Figure 3H). Similarly, results from IHC assay demonstrated that the level of CTBP2 immunoreactivity was highly correlated with PCIF1 in both local cohorts (Figure 3, I and J).

To determine the independent prognostic value of CTBP2 in HNSCC, we first analyzed TCGA data. The results showed conspicuously higher *CTBP2* expression in tumor tissues (Supplemental Figure 3A). The CTBP2 protein levels were also remarkably upregulated in HNSCC tissues from both cohorts (Supplemental Figure 3, B, C, I, and J). Importantly, a high CTBP2 protein level was significantly correlated with high tumor stage and tumor grade in cohort 1 (Supplemental Figure 3, D–G), whereas a high CTBP2 protein level was significantly correlated with T classification, tumor stage, and lymph node metastasis status in cohort 2 (Supplemental Figure 3, K–N). Meanwhile, there was a statistically significant difference in overall survival rates (*P* < 0.05) between patients with CTBP2-high and CTBP2-low tumors in both cohorts (Supplemental Figure 3, H and O).

PCIF1 is well known as a cap-specific *N*^6^-methyltransferase of m^6^Am (10–12). We wondered whether CTBP2 is involved in cap-adjacent m^6^Am deposition on mRNA. To test that, we used two sgRNAs targeting the *CTBP2* gene to generate two CTBP2-KO SCC25 cell lines. Western blot analysis showed that both sgRNAs led to efficient depletion of CTBP2 in cells (Figure 3K). To determine whether PCIF1 and CTBP2 affect the mRNA m^6^Am modification, we measured the level of m^6^Am in poly(A)-enriched RNA after decapping using liquid chromatography–tandem mass spectrometry. Excitingly, we found that KO of either PCIF1 or CTBP2 reduced m^6^Am level but not m^6^A level in SCC25 cells (Figure 3, L and M), suggesting that CTBP2 is involved in m^6^Am modification mediated by PCIF1.

Subsequently, we conducted functional validation of PCIF1 and CTBP2 using cellular models, wherein we established stable cell lines with PCIF1 knockdown and subsequently performed additional CTBP2 knockdown (Supplemental Figure 4A). Moreover, cell functional experiments targeting PCIF1 knockout and dual knockout of PCIF1 and CTBP2 were performed in HNSCC cell lines. The results revealed that the double knockout of PCIF1 and CTBP2 did not exhibit substantial alterations in the tumor cell phenotype compared with the sole knockout of PCIF1 (Supplemental Figure 4, B–G). These observations indicated the potential role of CTBP2 as a crucial auxiliary factor to PCIF1, exerting key molecular functional effects in HNSCC.

To further investigate the potential interaction between PCIF1 and CTBP2, we used cross-linking and immunoprecipitation followed by high-throughput sequencing (CLIP-Seq) to assess the similarity of the mRNAs bound by these proteins. By analyzing the PCIF1 and CTBP2 CLIP-Seq data sets obtained from the SCC25 cell line, we observed a high degree of overlap between the mRNAs bound by PCIF1 and CTBP2 (Supplemental Figure 5A). Interestingly, our analysis revealed similar enrichment patterns for PCIF1- and CTBP2-bound mRNAs. Both CTBP2 and PCIF1 exhibited enrichment in “cell migration” and “positive regulation of transcription” pathways (Supplemental Figure 5, B and C). Notably, we observed pronounced enrichment of these pathways in the co-bound mRNAs (Supplemental Figure 5D), confirming potential regulatory interplay between PCIF1 and CTBP2.

### TET2 transcript is a potential downstream target of PCIF1 and CTBP2 in HNSCC.

**Figure 4:**
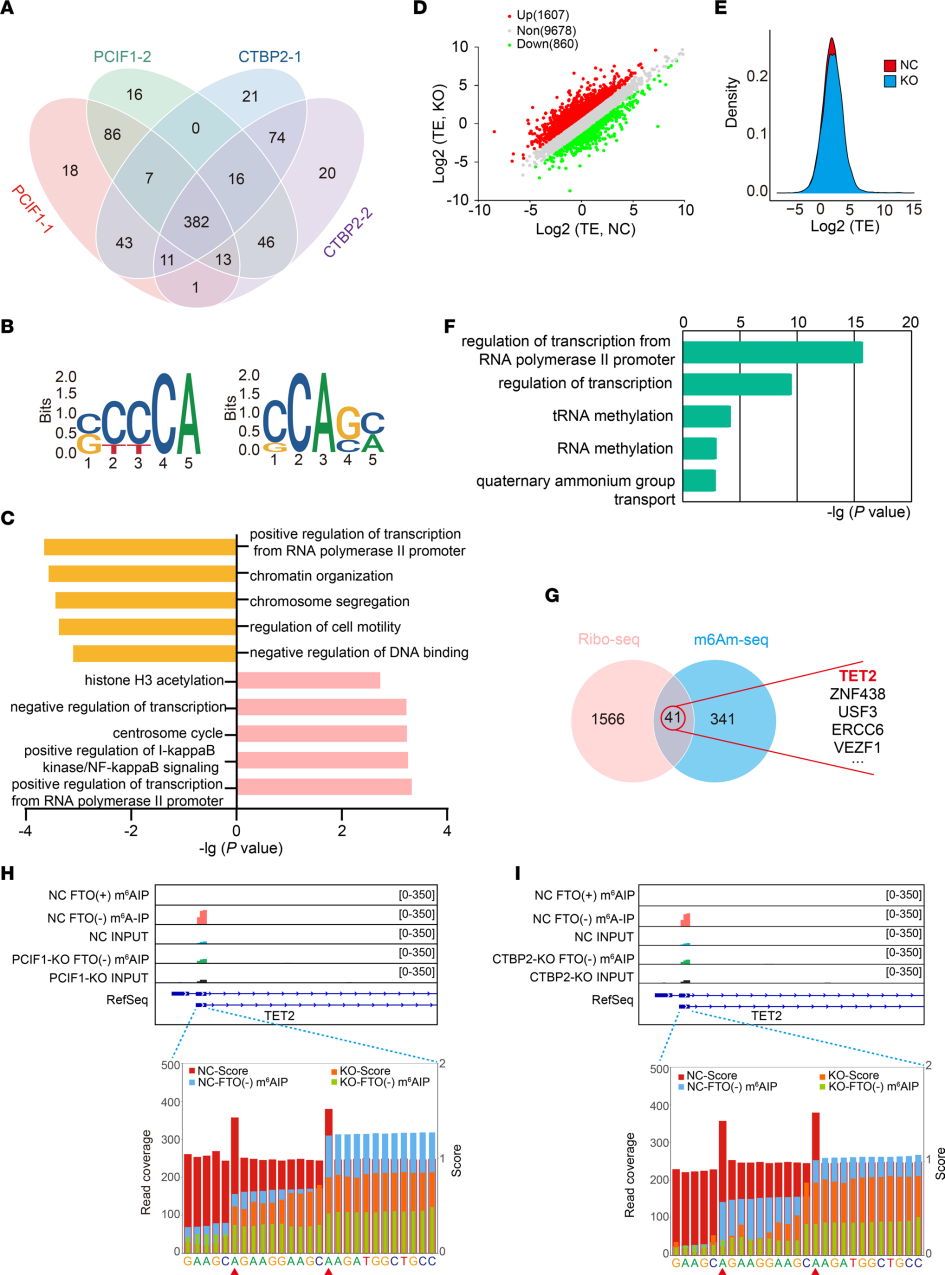
m^6^Am-Seq identifies TET2 as a target of CTBP2 and PCIF1. (**A**) Venn diagram shows the integration of PCIF1-dependent modified genes and CTBP2-dependent modified genes; 382 genes are consistently modified by PCIF1 and CTBP2. (**B**) The top consensus m^6^Am motif identified in SCC25 cells with or without PCIF1 KO (left) and SCC25 cells with or without CTBP2 KO (right). (**C**) Bar plots showing the top 5 GO terms (Biological Process, DAVID) for PCIF1-dependent modified genes (top) and CTBP2-dependent modified genes (bottom). (**D**) Scatterplot of the translation ratios (TRs) in PCIF1-WT and PCIF1-KO SCC25 cells. TRs were calculated by division of the ribosome-binding transcript signals by input RNA-Seq signals. The PCIF1-WT SCC25 cell group served as the NC group. (**E**) Cumulative distribution plot of the translation efficiency (TE) distribution in cells with or without PCIF1 KO. (**F**) Bar plots showing the top 5 GO terms of genes with increased TRs. (**G**) Venn diagram shows the intersection of genes in GO Biological Process terms (regulation of transcription) from genes with increased TRs (left) and PCIF1- and CTBP2-dependent modified genes (right). (**H** and **I**) Representative images of PCIF1-dependent modified (**H**) and CTBP2-dependent modified (**I**) single m^6^Am sites on the transcripts of TET2. The 2 adenosine residues with a high score (red bars) were defined as m^6^Am sites.

To identify the potential target transcripts modified by PCIF1 and CTBP2, we used PCIF1-KO and CTBP2-KO SCC25 cells for m^6^Am-Seq (15, 16), which allowed us to detect m^6^Am at single-base resolution. After rigorous quality control process and calculation, we identified a number of peaks ranging from 554 to 566 in replicates of PCIF1-KO or CTBP2-KO SCC25 cells (Figure 4A). Remarkably, we found that 382 m^6^Am-modified genes were shared among all samples, highlighting a commonality of transcripts modified by PCIF1 and CTBP2 (Figure 4A). Analysis of sequenced clusters identified the consensus “CA” motif within the m^6^Am sites for PCIF1 and CTBP2 (Figure 4B). Gene Ontology (GO) analysis demonstrated that the genes regulated by PCIF1 were substantially enriched in multiple biological processes, including regulation of transcription, regulation of IκB kinase/NF-κB signaling, centrosome cycle, and histone H3 acetylation pathways (Figure 4C). In addition, we found that a part of biological processes was jointly regulated by PCIF1 and CTBP2, such as regulation of transcription (Figure 4C).

Although the effects of cap-adjacent m^6^Am on mRNA are still debatable, recent studies suggested that the main function of m^6^Am is involved in negative regulation of cap-dependent translation of methylated mRNAs (11, 15). In light of this, we analyzed ribosome-protected mRNA fragments using ribosome sequencing (Ribo-Seq) to generate genome-wide translational landscapes in both control and PCIF1-KO SCC25 cells. We observed changes in ribosome footprints for a subset of transcripts (approximately 1,600 up- and 800 downregulated transcripts in Ribo-Seq) after PCIF1 KO (Figure 4, D and E). GO analysis revealed that most enriched categories were relevant to regulation of transcription and RNA methylation (Figure 4F). Upon Venn analysis of upregulated genes after PCIF1 KO in Ribo-Seq and 382 genes with m^6^Am peaks in m^6^Am-Seq, it was observed that 41 genes were overlapped in both gene sets (Figure 4G). Importantly, we found that *TET2*, a tumor suppressor gene that catalyzes the conversion of 5-methylcytosine (5mC) to 5-hydroxymethylcytosine (5hmC) and promotes DNA demethylation (17, 18), was the most upregulated gene among these 41 genes. Notably, analysis of our m^6^Am-Seq data identified 2 m^6^Am sites in the 5′-UTRs of *TET2* transcript in both PCIF1 KO and CTBP2 KO (Figure 4, H and I), suggesting that *TET2* might be a downstream target of PCIF1 and CTBP2 in HNSCC.

**Figure 5:**
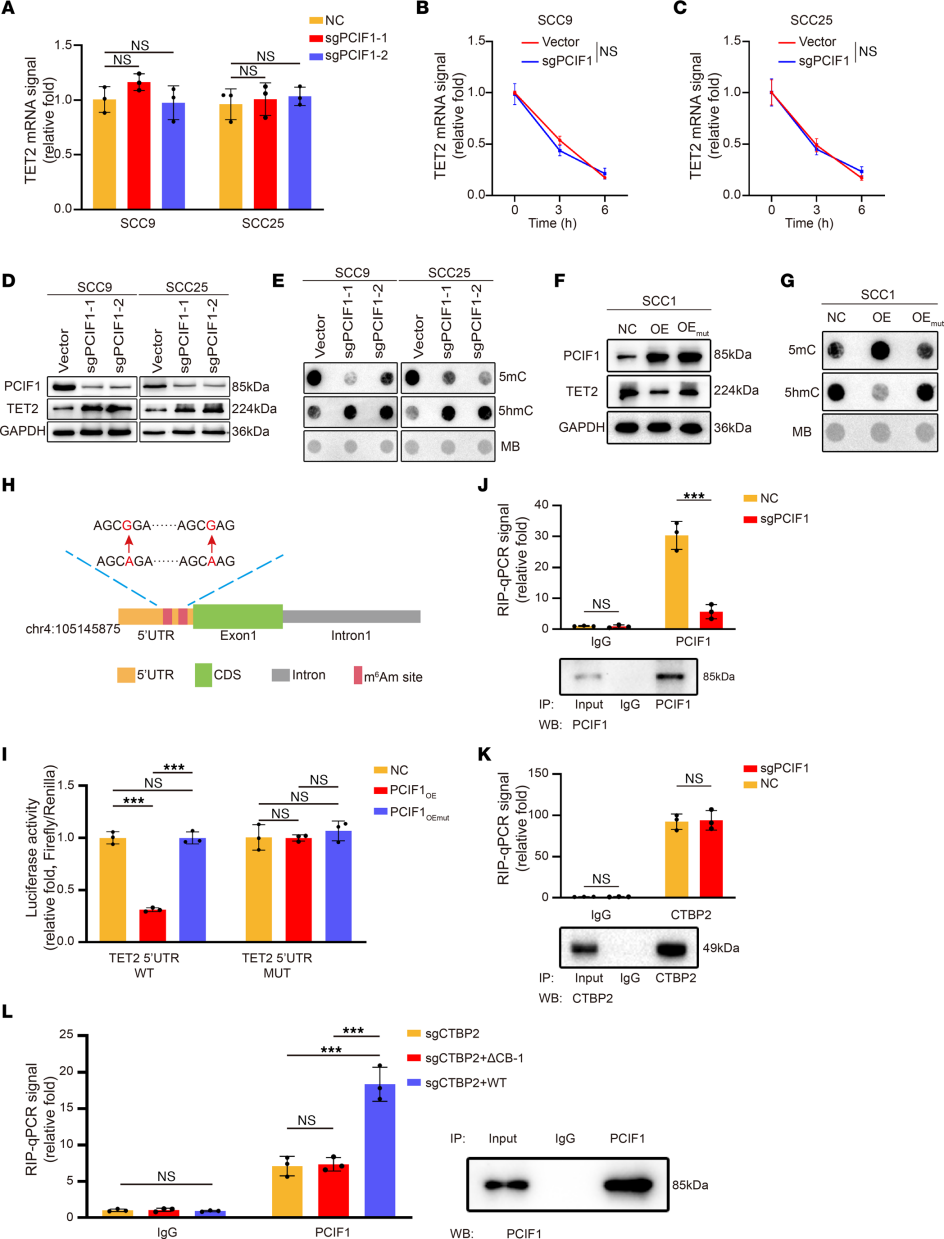
TET2 cap-adjacent m^6^Am modification suppresses the translation of *TET2* mRNA, and PCIF1 requires CTBP2 to bind *TET2* mRNA. (**A**) Real-time qPCR analysis of *TET2* mRNA expression in control and PCIF1-KO cells (*n* = 3). *P* > 0.05 by 1-way ANOVA with Tukey’s multiple-comparison test. (**B** and **C**) Real-time qPCR analysis of *TET2* mRNA levels at the indicated times in control and PCIF1-KO SCC9 (**B**) and SCC25 (**C**) cells after actinomycin D treatment (*n* = 3). *P* > 0.05 by 2-tailed unpaired Student’s *t* test. (**D** and **E**) TET2 expression (**D**) and DNA 5mC and 5hmC modification levels (**E**) were detected in control and PCIF1-KO cells. (**F** and **G**) TET2 expression (**F**) and DNA 5mC and 5hmC modification levels (**G**) were detected in SCC1 control cells and cells transfected with WT (OE) or mutant PCIF1 plasmid (OE_mut_). MB, Methylene blue. (**H**) Schematic diagram of TET2 5′-UTR m^6^Am site mutations. Red arrows denote A594G and A604G mutation. (**I**) Luciferase activity of TET2 5′-UTR WT or TET2 5′-UTR m^6^Am site mutation (5′-UTR MUT) in SCC1 control cells and cells transfected with WT (OE) or mutant PCIF1 plasmid (OE_mut_) (*n* = 3). *P* > 0.05, ***P < 0.001 by 1-way ANOVA with Tukey’s multiple-comparison test. (**J** and **K**) RNA immunoprecipitation (RIP)–qPCR analysis of *TET2* mRNA retrieved by anti-PCIF1 (**J**) and anti-CTBP2 (**K**) antibody in control and PCIF1-KO cells (*n* = 3). *P* > 0.05, ***P < 0.001 by 2-tailed unpaired Student’s *t* test. (**L**) RIP-qPCR analysis of *TET2* mRNA retrieved by anti-PCIF1 antibody in CTBP2-KO cells transfected with vector (sgCTBP2), PCIF1 binding–defective mutant of CTBP2 (ΔCB-1), and WT CTBP2 (*n* = 3). *P* > 0.05, ***P < 0.001 by 1-way ANOVA with Tukey’s multiple-comparison test.

Next, to test that possibility, the expression of *TET2* was determined by real-time PCR and Western blot analysis under different conditions. We found that the mRNA level of *TET2* was not altered after PCIF1 or CTBP2 KO, whereas the protein levels of TET2 were remarkably increased after PCIF1 KO (Figure 5, A and D, and Supplemental Figure 6A). We also evaluated *TET2* mRNA stability in control cells and in cells with PCIF1 or CTBP2 KO; data revealed that PCIF1 or CTBP2 depletion did not affect the *TET2* mRNA degradation rate (Figure 5, B and C, and Supplemental Figure 6, B and C). Moreover, we detected a dramatic decrease in global 5mC levels as well as a notable elevation in 5hmC levels in PCIF1-KO cells compared with control (Figure 5E). On the contrary, overexpression of WT PCIF1, but not mutant PCIF1, diminished *TET2* expression in SCC1 cells (Figure 5F). In addition, our dot blot data evidenced an increase in 5mC level in genomic DNA of WT PCIF1–overexpressing sample (Figure 5G). Conversely, 5hmC level appeared markedly decreased in the genome of the WT PCIF1 group compared with other groups (Figure 5G).

### Cap-adjacent m^6^Am of TET2 impedes the translation of TET2 mRNA.

Based on our results, modulation of *TET2* expression might occur in an m^6^Am-dependent manner. To test that, we cloned a 5′-UTR of *TET2* or the same region with mutated m^6^Am motif into luciferase reporter to find out the function of these m^6^Am modifications in gene regulation (Figure 5H). We found that overexpression of PCIF1 impaired the translation of luciferase reporters, while m^6^Am motif mutation or mutant PCIF1 abolished the translation attenuation by PCIF1, suggesting that these m^6^Am modifications were critical for PCIF1-mediated *TET2* expression (Figure 5I).

In searching for the detailed mechanism of the *TET2* m^6^Am modifications by PCIF1 and CTBP2, we wondered whether CTBP2 influences interactions of *TET2* mRNA with PCIF1, or vice versa. RNA immunoprecipitation–quantitative PCR assays were conducted to determine the interaction between PCIF1 or CTBP2 and cap-adjacent m^6^Am in *TET2* transcript. Our data showed that PCIF1 remarkably enriched *TET2* mRNA, while this relative enrichment (immunoprecipitated vs. input) was significantly suppressed in PCIF1-KO and CTBP2-KO cells (Figure 5, J and K, and Supplemental Figure 6, D and E). We also detected occupancy of CTBP2 on *TET2* mRNA (Figure 5, J and K, and Supplemental Figure 6, D and E). Surprisingly, depletion of PCIF1 showed no effect on the binding of CTBP2 to *TET2* mRNA (Figure 5, J and K, and Supplemental Figure 6, D and E), suggesting that CTBP2 is required for the interaction of PCIF1 and mRNA. In addition, rescue experiments were conducted using WT CTBP2 and a CTBP2 mutant with defective PCIF1 binding (ΔCB-1). The results demonstrated that WT CTBP2 effectively restored the binding of *TET2* mRNA in CTBP2-KO cells, while the mutant lacking PCIF1 binding failed to achieve this restorative effect (Figure 5L). This further validated the interaction between PCIF1 and CTBP2 and highlights the crucial role of CTBP2 in facilitating their binding to *TET2* mRNA.

To investigate whether the effects of PCIF1 on HNSCC are mediated by *TET2* activation, we knocked down *TET2* expression in WT or PCIF1-KO SCC9 and SCC25 cell lines by transfections with a ssiRNA targeting *TET2* (Supplemental Figure 7A). Consistently with our sequencing results, PCIF1 KO led to elevated levels of TET2 expression in SCC9 and SCC25 cells (Supplemental Figure 7A). In addition, we noticed a marked reduction in global 5mC levels in PCIF1-KO cells compared with controls, accompanied by a marked increase in 5hmC levels in both cell lines (Supplemental Figure 7A), and increase of 5hmC levels was abrogated by the knockdown of TET2 expression (Supplemental Figure 7A). Moreover, sgPCIF1-mediated repression of cell proliferation was relieved by the knockdown of TET2 expression, while PCIF1-mediated inhibition of migratory and invasive capability was partially rescued by TET2 knockdown (Supplemental Figure 7, B–D and F). At the same time, the apoptotic effects of PCIF1 were overturned by the knockdown of TET2 expression (Supplemental Figure 7E).

### PCIF1 and CTBP2 are required for HNSCC development in a carcinogen-induced mouse model.

**Figure 6:**
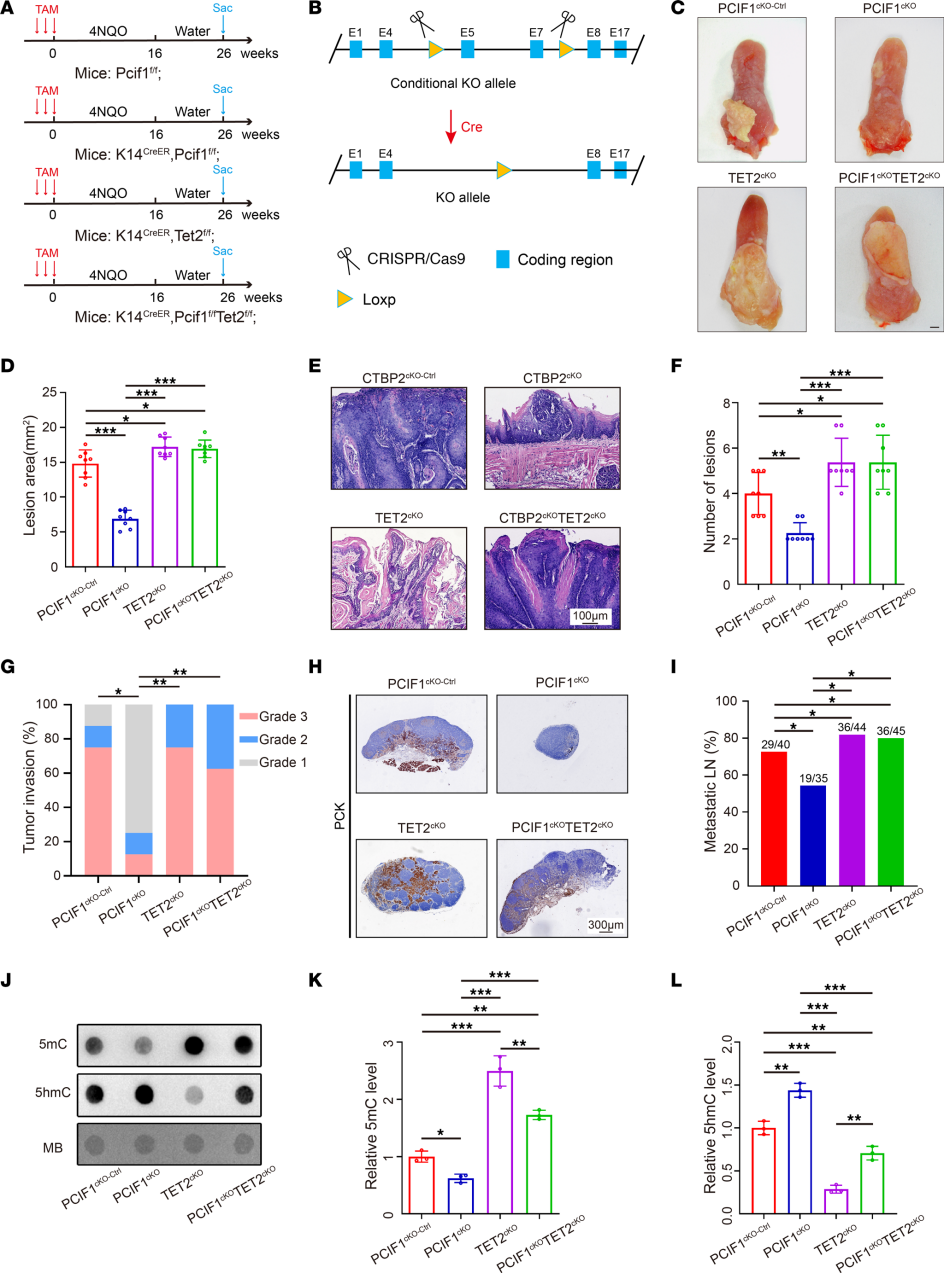
Conditional knockout of Pcif1 inhibits tumor growth and metastasis in vivo via enhanced expression of Tet2. (**A**) Experimental design of the carcinogen-induced HNSCC mouse model. Sac, sacrifice. (**B**) Diagram of Cre-dependent conditional knockout strategy for Pcif1. (**C**) Representative image of visible tongue lesions in the indicated groups. Scale bar: 1 mm. (**D**) Quantification of HNSCC lesion area in the indicated groups (*n* = 8). *P < 0.05, ***P < 0.001 by 1-way ANOVA with Tukey’s multiple-comparison test. (**E**) Representative H&E staining of HNSCC in the indicated groups. Scale bar: 100 μm. (**F**) Quantification of HNSCC number of lesions in the indicated groups (*n* = 8). *P < 0.05, **P < 0.01, ***P < 0.001 by 1-way ANOVA with Tukey’s multiple-comparison test. (**G**) Quantification of HNSCC tumor grade in the indicated groups. *P < 0.05, **P < 0.01 by Pearson’s χ^2^ test. (**H**) Representative PCK staining of metastatic lymph node in the indicated groups. Scale bar: 300 μm. (**I**) Quantification of metastatic lymph node percentage in the indicated groups. *P < 0.05 by Pearson’s χ^2^ test. (**J**–**L**) Representative dot blot image (**J**) and quantitative analysis of DNA 5mC (**K**) and 5hmC (**L**) modification levels in the indicated groups (*n* = 3). *P < 0.05, **P < 0.01, ***P < 0.001 by 1-way ANOVA with Tukey’s multiple-comparison test.

Toward this end, we wanted to examine whether our findings could be validated in vivo by using a 4NQO-induced mouse HNSCC model, which closely resembles human HNSCC (19) (Figure 6A). First, mice harboring the floxed allele (*Pcif1^fl^*) were mated to a *K14^CreER^* strain to obtain homozygous *K14^CreER^* *Pcif1^fl/fl^* conditional knockout mice (Pcif1-cKO) (Figure 6B). To generate oral epithelium–specific *Tet2* knockout or rescue mice, the *Tet2^fl^* mice were crossed to *K14^CreER^* *Pcif1^fl/fl^* mice to generate *K14^CreER^* *Tet2^fl/fl^* conditional knockout mice (Tet2-cKO) and *K14^CreER^* *Pcif1^fl/fl^* *Tet2^fl/fl^* double-knockout mice (Pcif1-Tet2-dkO). Briefly, control, Pcif1-cKO, Tet2-cKO, and Pcif1-Tet2-dkO mice at 6 weeks of age were given 4NQO in the drinking water for 16 weeks and an additional 10 weeks of normal drinking water before harvesting (Figure 6A).

Tongue and cervical lymph node tissues were collected from each mouse for histological and biochemical analysis. We found that macroscopic oral lesion area and number were less prominent in the Pcif1-cKO group as compared with the control group (Figure 6, C–F), while Tet2-cKO mice demonstrated a moderate increase in lesion area and number (Figure 6, C–F). Remarkably, genetic ablation of *Tet2* was able to enhance the formation of HNSCC (Figure 6, C–F). Histological examination showed that loss of Pcif1 reduced the frequency of higher-grade HNSCC according to our previous published criteria (20) (Figure 6, E and G). However, Pcif1-Tet2-dkO restored the aggressiveness of 4NQO-induced HNSCC to control mouse levels (Figure 6, E and G). IHC staining also confirmed that Pcif1 was significantly decreased in tumors derived from Pcif1-cKO and Pcif1-Tet2-dkO mice compared with control mice (Supplemental Figure 8, A and B). At the same time, IHC staining revealed depletion of Tet2 expression in Tet2-cKO and Pcif1-Tet2-dkO mice, whereas Pcif1-cKO mice displayed an elevated level of Tet2 compared with WT mice (Supplemental Figure 8, C and D). As anticipated, expression of the proliferation marker Ki67 was lower in tumors derived from Pcif1-cKO mice compared with control mice (Supplemental Figure 8, E and F). Anti–pan-cytokeratin (anti-PCK) staining revealed that Pcif1 deletion alone significantly reduced lymph node metastasis, but in Tet2-cKO and Pcif1-Tet2-dkO mice, the lymph node metastasis rate was significantly increased (Figure 6, H and I). While depletion of Pcif1 dramatically increased global 5hmC levels, the blockage of Tet2 generated markedly less 5hmC (Figure 6, J–L).

To validate our findings, we also used a TET enzyme inhibitor, Bobcat339 (BC339), to treat mice bearing HNSCC. For this, mice were given 4NQO with or without BC339 in the drinking water (Supplemental Figure 9A). Similarly, we found that treatment of BC339 reversed the effects of PCIF1 KO on HNSCC formation and aggressiveness (Supplemental Figure 9, B–F). Moreover, reduction of cell proliferation and metastasis in Pcif1-cKO samples was greatly rescued by treatment with BC339 (Supplemental Figure 9, G–J). The level of global 5hmC was recovered after BC339 treatment (Supplemental Figure 9, K–M).

**Figure 7:**
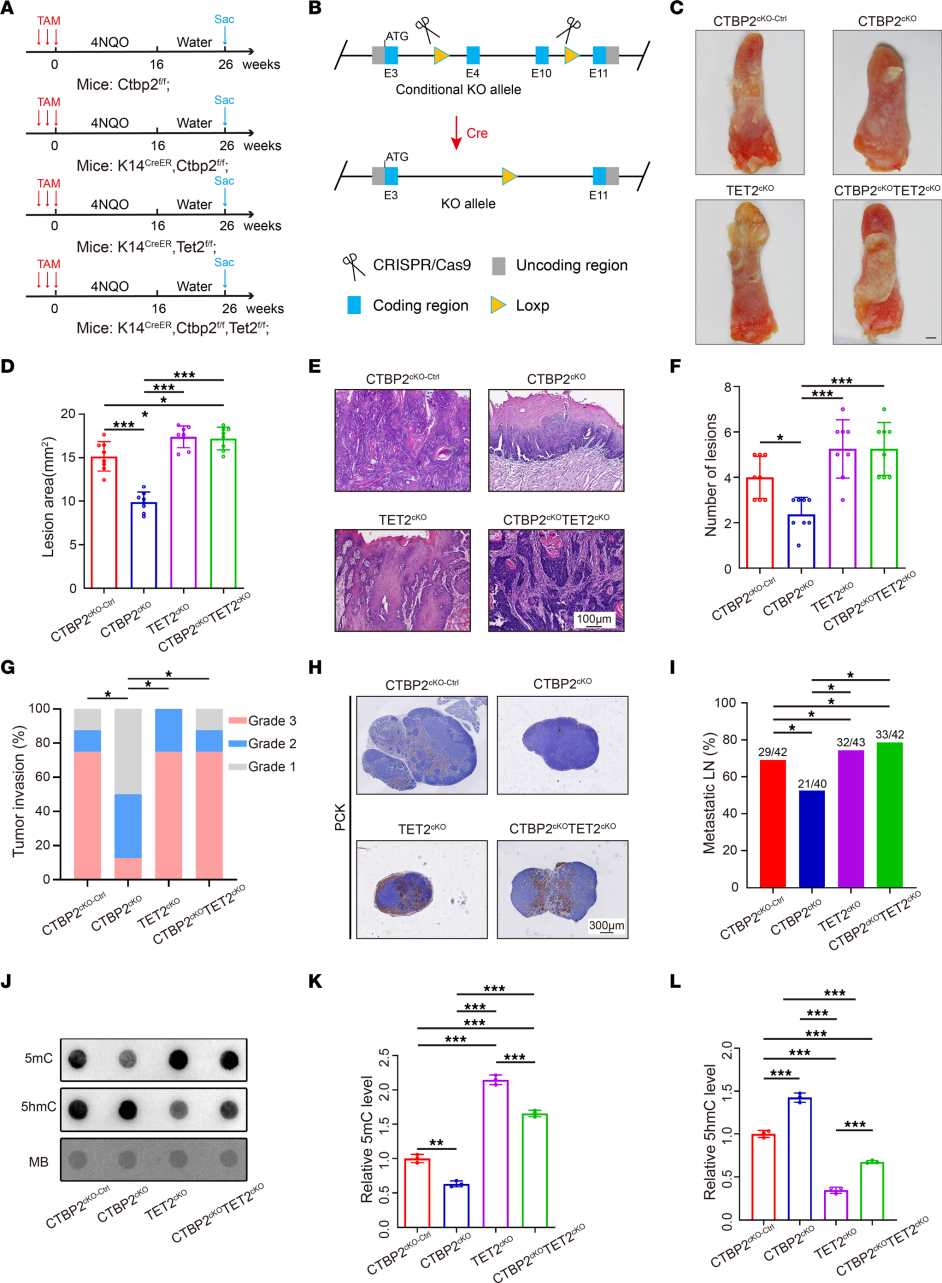
Conditional knockout of CTBP2 inhibits tumor growth and metastasis in vivo via enhanced expression of TET2. (**A**) Experimental design of the carcinogen-induced HNSCC mouse model. (**B**) Diagram of Cre-dependent conditional knockout strategy for Ctbp2. (**C**) Representative image of visible tongue lesions in the indicated groups. Scale bar: 1 mm. (**D**) Quantification of HNSCC lesion area in the indicated groups (*n* = 8). *P < 0.05, ***P < 0.001 by 1-way ANOVA with Tukey’s multiple-comparison test. (**E**) Representative H&E staining of HNSCC in the indicated groups. Scale bar: 100 μm. (**F**) Quantification of HNSCC number of lesions in the indicated groups (*n* = 8). *P < 0.05, ***P < 0.001 by 1-way ANOVA with Tukey’s multiple-comparison test. (**G**) Quantification of HNSCC tumor grade in the indicated groups. *P < 0.05 by Pearson’s χ^2^ test. (**H**) Representative PCK staining of metastatic lymph node in the indicated groups. Scale bar: 300 μm. (**I**) Quantification of metastatic lymph node percentage in the indicated groups. *P < 0.05 by Pearson’s χ^2^ test. (**J**–**L**) Representative dot blot image (**J**) and quantitative analysis of DNA 5mC (**K**) and 5hmC (**L**) modification levels in the indicated groups (*n* = 3). **P < 0.01, ***P < 0.001 by 1-way ANOVA with Tukey’s multiple-comparison test.

We then proceeded to test the function of *Ctbp2* in vivo. Since mice with *Ctbp2* whole-body knockout are embryonic lethal (21), we then generated a conditional-knockout mouse in which critical exons of the *Ctbp2* gene are flanked with *loxP* sites (*Ctbp2^fl^*) (Figure 7B). *Ctbp2^fl/fl^* mice do not show any abnormality and are fertile (data not shown). *K14^CreER^* mice were crossed with *Ctbp2^fl/fl^* mice to obtain *K14^CreER^* *Ctbp2^fl/fl^* (Ctbp2-cKO) mice for characterizing the role of Ctbp2 in HNSCC tissues. Notably, the oral epithelium of Ctbp2-cKO mice appeared normal macroscopically and histologically after administration of tamoxifen (data not shown). We then applied a similar strategy to study the curative effect of *Ctbp2* in murine HNSCC (Figure 7A). We found that tongue tissues of Ctbp2-cKO exhibited less lesion area and fewer lesions (Figure 7, C–F). The tumor grade of Ctbp2-cKO HNSCC was not as advanced as compared with the control (Figure 7G). Compared with control mice, the mutant mice showed weakened proliferative and metastatic abilities of HNSCC cells (Supplemental Figure 10, E and F, and Figure 7, H and I). These results indicated that Ctbp2 is essential in a chemically induced HNSCC mouse model. And it is notable that *Tet2* knockout in Ctbp2-cKO mice can compensate for *Ctbp2* loss in terms of tumor progression. In Tet2-cKO and Ctbp2-Tet2-dKO mice, HNSCC lesion area and lesion number were significantly increased in comparison with Ctbp2-cKO mice (Figure 7, D and F). Moreover, Tet2-cKO and Ctbp2-Tet2-dKO also restored invasiveness of HNSCC (Figure 7G). Both Ctbp2-cKO and Ctbp2-Tet2-dKO mice showed a marked reduction in Ctbp2 IHC staining (Supplemental Figure 10, A and B). IHC staining confirmed that Tet2 protein level was significantly elevated in tumors derived from Ctbp2-cKO and Ctbp2-Tet2-dkO mice compared with control mice (Supplemental Figure 10, C and D). We also examined the role of Tet2 in the development of Ctbp2-cKO mice. Tumors stained with Ki67 and cervical lymph node stained with PCK showed enhanced proliferative and metastatic abilities in comparison with control mice (Supplemental Figure 10, E and F, and Figure 7, H and I). Blocking Tet2 significantly reduced the amount of global 5hmC produced, but depletion of Ctbp2 significantly increased it (Figure 7, J–L).

## Discussion

The importance of epitranscriptomic modification of RNA in tumorigenesis and metastasis of tumors is well appreciated in HNSCC and many other cancers (6, 22, 23). As one of the most prevalent RNA modifications in mRNAs, m^6^Am and the methyltransferase PCIF1 have been linked to cancer progression and therapeutic response across different cancer types. Depletion of PCIF1 in colorectal cancer cells enhances the efficacy of anti–PD-1 treatment by modulating immune response factors and promoting tumor-infiltrating natural killer cell recruitment (24). In gastric cancer, PCIF1 suppresses *TM9SF1* mRNA translation, contributing to tumor aggressiveness and metastasis (1). Another study demonstrated that PCIF1 exerts suppressive effects on glioma growth and survival, which may not entirely depend on its methyltransferase activity (25). Yet the role of m^6^Am modification in HNSCC development and the detailed mechanism underlying this modification in HNSCC remain undetermined. Our study consistently demonstrated the interaction between PCIF1 and CTBP2, supported by robust MS-iTRAQ results and protein interactions assays. This finding contrasts with a previous study (26), highlighting the influence of different experimental techniques and methodologies used. Additionally, different cell types (SCC25 cells in our study compared with HEK293T cells in the previous study) may contribute to divergent results, emphasizing the importance of considering cellular context in protein-protein interaction studies. In the current study, we uncovered a regulatory mechanism of m^6^Am deposition on mRNA mediated by a PCIF1-CTBP2 complex. Combining in vitro and in vivo assays, we demonstrated that PCIF1-CTBP2 catalyzed cap m^6^Am modification on *TET2* transcript and negatively regulated its translation. These findings highlight a functional role of PCIF1 and m^6^Am in HNSCC progression and provide evidence supporting the development of novel therapeutic strategies for HNSCC treatment.

Although dynamic and reversible m^6^Am RNA modification on the mRNA cap has been well characterized, the functions of m^7^G cap–adjacent m^6^Am have not reached a consensus. Several groups using different models suggested that m^6^Am level is associated with the stability of m^6^Am-modified mRNAs (12, 27). On the other hand, other researchers reported that cap-specific m^6^A promotes translation of mRNAs starting from m^6^Am based on a transcriptome-wide analysis (10). However, recent studies found that m^6^Am modification negatively impacts cap-dependent translation of methylated mRNAs (1, 11). In our study, the percentage of cap-adjacent m^6^Am–modified genes that had translation changes was relatively low (10.7%, 41/382). However, based on previous publications, this seems to be a common issue of cap-adjacent m^6^Am. For example, Wang et al. reported that 172 of 2,811 m^6^Am-marked genes showed downregulated transcription (28). In our study, there is a lack of evidence supporting that m^6^Am modification is involved in the stability of mRNA. Rather, it inhibits the translation efficiency of target mRNA with m^6^Am modification. This discrepancy may be explained by different methods used in different studies. Hence, different functions attributed to m^6^Am require more systematic examination and clarification.

The deposition of RNA modification often requires the corroboration of multiple proteins. For example, RNA m^6^A modification is catalyzed by protein complex formed by methyltransferase like 3 (METTL3), METTL14, and Wilms’ tumor 1–associating protein (WTAP) (29). Moreover, METTL1 interacts with WD repeat–containing protein 4 (WDR4) to mediate m^7^G transfer RNA modifications to promote mRNA translation (22). Here, we report that CTBP2 can interact with PCIF1 to regulate m^6^Am deposition in the cap region of mRNA. Normally, CTBP2 acts as a transcriptional corepressor and exerts its function via recruitment of histone deacetylases and histone methyltransferases (21). Besides, CTBP2 is often localized in the nuclear region owing to a nuclear localization signal in its N-terminal region (30). In this case, CTBP2 plays a key role in the localization of pre-mRNAs into the nucleus, where it is enriched with pre-mRNA processing factors and the catalytic activity of the m^6^Am methyltransferase. In HNSCC, microRNA-133a blocks the expression of CTBP2 and suppresses the tumorigenic ability of HNSCC cell lines (31). However, the exact role of CTBP2 in HNSCC has not been fully explored. Since the global deficiency of CTBP2 manifests embryonic lethality (21), we generated a *Ctbp2* conditional knockout mouse model to study the in vivo function of CTBP2. Interestingly, the in vivo model demonstrated that CTBP2 is essential for spontaneous HNSCC development and progression.

Although various transcripts were m^6^Am modified, it seems that *TET2* is a major downstream mediator of the PCIF1-CTBP2 complex based on our functional studies. *TET2* is well recognized as a transcriptional regulatory factor and tumor suppressor in numerous tumors (32–35). Loss-of-function mutations of the *TET2* gene are commonly detected in hematopoietic tumors, such as acute myeloid leukemia, chronic myelomonocytic leukemia, and peripheral T cell lymphomas (36). *TET2* mutation resulted in a reduced global level of genomic 5hmC and a progressive increase of the hematopoietic stem cell compartment (37). Furthermore, downregulation of TET2 expression was frequently perceived in HNSCC samples, while restoration of TET2 deficiency can repress cell proliferation, migration, and chemoresistance in HNSCC cells (18). The repression of TET2 might reflect more diverse transcriptional regulation, which is required for tumorigenic properties of cancer cells. Here, we report the epitranscriptomic mechanism of how TET2 could be regulated in cancer, providing a rationale for developing therapeutic strategies for TET2 intervention.

In conclusion, our studies demonstrated that both PCIF1 and CTBP2 were independent predictors in patients with HNSCC, and patients with increased nuclear expression of PCIF1 and CTBP2 in tumor lesions tended to have an unfavorable prognosis. PCIF1 and CTBP2 form a protein complex that is required for catalyzing cap m^6^Am modification. Blocking the PCIF1-CTBP2 complex impaired the tumorigenic abilities of tumor cells partially as a result of activated translation of *TET2* tumor suppressor. Our results suggest that the PCIF1-CTBP2 complex may serve as a promising target for anticancer treatment.

## Methods

### Human samples.

A cohort containing 81 HNSCC specimens and 57 adjacent normal tissue samples was obtained from the Department of Oral and Maxillofacial Surgery, The First Affiliated Hospital of Sun Yat-sen University. Another cohort comprising 40 HNSCC specimens and 40 adjacent normal tissue samples was obtained from the Hospital of Stomatology, Sun Yat-sen University.

### Transgenic mice.

Six-week-old male and female C57BL/6 mice were used for animal experiments. *K14^CreER^* mouse strains were purchased from The Jackson Laboratory. *Pcif1^fl^* mice were purchased from Biocytogen Pharmaceuticals. *Ctbp2^fl^* mice were purchased from GemPharmatech. *Tet2^fl^* mice were gifts from Yufeng Liu (Zhujiang Hospital, Southern Medical University, Guangzhou, China). For HNSCC induction, mice aged 6 weeks received drinking water containing 50 μg/mL 4NQO (Sigma-Aldrich, N8141) for 16 weeks and were fed with normal drinking water for another 10 weeks. For conditional deletion of *Pcif1*, *Ctbp2*, or *Tet2* in mice, tamoxifen (Sigma-Aldrich, T5648) was first dissolved in pure ethanol and diluted with corn oil (Aladdin, C116025) to produce a 10% ethanol/tamoxifen/corn oil mixture at 20 mg/mL. Cre was activated by intraperitoneal injection of 100 μL tamoxifen for 3 consecutive days to induce recombination. For TET2 inhibitor treatment, Bobcat339 hydrochloride (MedChemExpress, HY-111558A; 20 mg/mL) was fed to mice along with 4NQO for 16 weeks.

### Cell culture and transfection.

HOK cells were purchased from ScienCell. SCC1 was obtained from the laboratory of T. Carey (University of Michigan, Ann Arbor, Michigan, USA). HEK293T, SCC9, SCC15, and SCC25 were purchased from the American Type Culture Collection, and UM1 was provided by Xiaofeng Zhou (Center for Molecular Biology of Oral Diseases, Department of Periodontics, College of Dentistry, University of Illinois at Chicago, Chicago, Illinois, USA). All cell lines were cultured with Dulbecco’s modified Eagle/F12 medium (DMEM/F12, Gibco, C11330500BT) with 10% FBS (Gibco, 10270-106) and 1% penicillin/streptomycin (Gibco, 15140-122) in a 37°C incubator containing 5% CO_2_.

Small guide RNA targeting the *PCIF1* or *CTBP2* gene was cloned into LentiCRISPRv2 (Addgene) to knock out PCIF1 or CTBP2. For PCIF1 overexpression plasmids, the full-length open reading frames of the human *PCIF1* gene (NCBI Reference Sequence NM_022104.3) were cloned into the pLVX vector (GeneCopoeia). The PCIF1 catalytic inactive mutant (aa160–163, LFPD to AFPA) was provided by Tianhua Zhou (Department of Cell Biology and Department of Gastroenterology, Sir Run Run Shaw Hospital, Zhejiang University School of Medicine, Hangzhou, China). For knockdown of TET2, cells were transfected with control or TET2 siRNA oligonucleotides. The oligonucleotide sequences are provided in Supplemental Table 4.

For mapping of CTBP2 domains that interact with PCIF1, we obtained various truncated CTBP2 proteins with HA tag into pRP vector and cotransfected them with pLVX-FLAG-PCIF1 into HEK293T using Lipofectamine 2000 reagent (Gibco, 25200072). Twenty-four to forty-eight hours later, cell lysate was immunoprecipitated with anti-FLAG antibody (Cell Biological, RM1002) and Protein A/G magnetic beads (Thermo Fisher Scientific, 88803). The CTBP2 truncated proteins were detected with anti-HA antibody (Abcam, ab9110) after immunoblotting.

### Western blotting.

Western blotting was performed as described previously (22). In brief, protein lysates were isolated with radioimmunoprecipitation assay buffer (Beyotime) containing proteinase inhibitor cocktail (Roche, Shanghai, China), subjected to 10% SDS-PAGE, and electrotransferred to the polyvinylidene fluoride membrane. The membrane was blocked with 5% skimmed milk and incubated with primary anti-PCIF1 (Cell Signaling Technology, 98085S), anti-GAPDH (Proteintech, 10494), anti-CTBP2 (Proteintech, 10346), and anti-TET2 (Abcam, ab94580). Then the membrane was incubated with anti-rabbit secondary antibodies (Proteintech, SA00001-2). Protein levels were detected using a Tanon 5200 Multi intelligent imaging system (Tanon, China).

### Cell proliferation assay and colony formation assay.

Cell proliferation was conducted with Cell Counting Kit-8 (CCK8) (Dojindo) following the manufacturer’s protocol. Cell suspension was added into 96-well plates at a density of 1,000 cells per well. The CCK8 reagent was added, and the absorbance at 490 nm was measured by microplate reader (Tecan, Infinite m200 PRO). For the colony formation experiments, cells were plated into 6-well plates (500 cells per well) in DMEM/F12 with 10% FBS for 14 days. The colonies were fixed with 4% paraformaldehyde, stained with crystal violet, and counted.

### Cell migration and invasion assay.

For migration assay, cell suspension was suspended in 200 μL FBS-free DMEM/F12 culture medium and added into a Transwell insert (Corning, China) at a density of 5 × 10^4^ cells per well, and 500 μL of DMEM/F12 containing 1% FBS was added to the lower compartment. For invasion assay, the chambers were precoated with 5% Matrigel (BioCoat, China). Then the cells were added as described for the migration assay. After 48 or 72 hours, the chambers were collected and stained by crystal violet, followed by quantification under a microscope in 5 random fields.

### Cell apoptosis and cell cycle assay.

The Annexin V–FITC/PI Apoptosis Assay Kit (MultiSciences) and cell cycle staining kit (MultiSciences) were used according to the manufacturer’s protocol. The fixed and stained cells were analyzed by flow cytometry using CytoFLEX (Beckman). Apoptotic cells consisted of early apoptotic cells and late apoptotic cells. FlowJo software version 7.6 (FlowJo LLC) was used for data analysis.

### Coimmunoprecipitation assays.

Coimmunoprecipitation was performed to investigate the binding protein with PCIF1. Cells were lysed with IP buffer (Beyotime, P0013) by incubation on ice for 30 minutes, followed by centrifugation at 10,000*g* for 10 minutes at 4°C. Parts of the supernatant were removed as input. The remaining cleared supernatant was incubated with magnetic beads at 4°C with gentle rotation for 30 minutes to remove nonspecific proteins, followed by transfer of the supernatant with a magnetic frame. Then, 2 μg primary antibody was added to the precleared lysate for 2 hours with gentle rotation at 4°C. Pretreated magnetic beads were added to the above samples with incubation at room temperature for 1 hour with gentle rotation, followed by adsorption of the magnetic beads with a magnetic frame and removal of the supernatant. Then the beads were resuspended with IP buffer 3 times, and 2× SDS sample buffer was added to the beads. After boiling of the samples at 100°C for 5 minutes, the boiled samples were transferred to SDS-PAGE gel.

### Liquid chromatography–tandem mass spectrometry.

To identify the cofactor with PCIF1, liquid chromatography–tandem mass spectrometry (LC-MS/MS) was performed with coimmunoprecipitation samples. Proteins were separated using SDS polyacrylamide gels, followed by staining with Coomassie blue to visualize protein bands. The gel bands with proteins were cut and put into a centrifuge tube. Then gel bands were rinsed with ddH_2_O, decolorized with decolorizing solution (50% acetonitrile, Thermo Fisher Scientific, A998-4L; and 25 mM ammonium bicarbonate, Thermo Fisher Scientific, 1066-33-7) to completely white with acetonitrile, and vacuum-dried. Next, dithiothreitol (DTT; Amresco, 0281-BEJ-100G) was added to the tube at 56°C for 1 hour. After removal of DTT, the reduced protein was alkylated by iodoacetamide (Sigma-Aldrich, I6125-10G) and incubated at room temperature in the dark for 45 minutes. Then ammonium bicarbonate and acetonitrile were used for cleaning and decolorization. The dried gel bands were digested with trypsin at 37°C overnight. Subsequently, formic acid (Thermo Fisher Scientific, 64-18-6) was added to stop the digestion reaction and detected by Q Exactive Mass Spectrometer (Thermo Fisher Scientific).

### RNA extraction and quantitative real-time PCR.

Total RNAs were extracted with TRIzol reagent (Invitrogen) according to the manufacturer’s instruction. Reverse transcription was conducted with 1 μg RNA using PerfectStart Green qPCR SuperMix (TransGen Biotech, AQ601). Then quantitative PCR (qPCR) was carried out in a StepOnePlus Real-Time PCR Instrument (Thermo Fisher Scientific). The relative mRNA expression levels were calculated using GAPDH as the internal control. The primer sequences used in this study are provided in Supplemental Table 4.

### Actinomycin D assay.

Cells were treated with actinomycin D (MedChemExpress, HY-17559), and RNA was obtained at the indicated times. *TET2* mRNA stability was detected by real-time qPCR assay. The mRNA level at the start time point was used for normalization.

### CLIP-Seq.

The CLIP-Seq Kit (BersinBio) was used according to the manufacturer’s protocol. Before cross-linking, 4-thiouridine (Macklin, 13957-31-8) was supplemented to the cell culture medium at a final concentration of 100 μM to facilitate the cross-linking of RNA and proteins. Following a wash with prechilled 1× PBS, the cells were exposed to 365 nm UV light for 10 minutes at a dosage of 0.15 J/cm^2^. Subsequently, the cells were meticulously harvested and subjected to centrifugation for supernatant removal. The lysed cells were then subjected to digestion using a cell lysis buffer and RNase T1, and 100 μL of the resulting supernatant was collected as the input sample. The remaining samples were incubated overnight with either anti-CTBP2 (Cell Signaling Technology, 13256S), anti-PCIF1 (Abcam, ab271081), or IgG antibodies. Immunoprecipitation (IP) was performed using Protein A/G magnetic beads, followed by sequential washing steps. DNase I digestion and bead washing were subsequently carried out, after which protein digestion was performed, and the supernatant from the immunoprecipitated samples was collected. Finally, both the input and immunoprecipitated samples’ supernatants were subjected to protein digestion to prepare the RNA samples for subsequent high-throughput sequencing analysis.

### m^6^Am-Seq.

Total RNA extracted from SCC25 cells with PCIF1 KO or CTBP2 KO and SCC25 control cells was fragmented by RNA fragmentation reagents (New England Biolabs). Ten nanograms of the fragmented RNA was used as input sample. The specific anti-m^7^G antibody (Medical & Biological Laboratories, RN017M) was preincubated with Pierce protein A/G magnetic beads at 4°C for 4 hours. The fragmented RNA was incubated with the antibody-beads at 4°C overnight. Then RNA fragments with m^7^G were obtained by phenol-chloroform extraction, and 1 ng RNA fragments with m^7^G was used as m^7^G-IP sample. The rest of the RNA was subjected to m^6^Am demethylation treatment by FTO (active motif, 31572) at 37°C for 30 minutes or treated as a control group. The demethylated RNA was collected by another phenol-chloroform extraction. The obtained demethylated RNA and control RNA were subjected to m^6^A immunoprecipitation with the specific anti-m^6^A antibody (Synaptic Systems, 202003). The RNA was incubated with pre-bound antibody-beads at 4°C overnight. After washing and phenol-chloroform extraction, the obtained RNA sample was used as FTO^+^ m^6^A-IP and FTO^–^ m^6^A-IP samples. All the input, m^7^G-IP, FTO^+^ m^6^A-IP, and FTO^–^ m^6^A-IP samples were subjected to library construction using SMARTer Stranded Total RNA-Seq Kit v2 - Pico Input Mammalian (Takara-Clontech, Japan, 634413) according to the manufacturer’s protocol. The libraries were sequenced on MGISEQ-2000 with PE150.

### Identification of PCIF1-dependent and CTBP2-dependent m^6^Am site.

An m^6^Am peak was identified as described previously (15). The peak was highly enriched in m^6^A-IP sample and was decreased in FTO^+^ m^6^A-IP sample compared with FTO^–^ m^6^A-IP sample. To identify the m^6^Am site, m^6^Am score was defined for each nucleotide within the m^6^Am peak. The m^6^Am score considers normalized sequencing start rate in untreated samples m1 = (FTO^–^ start reads) / (FTO^–^ depth) and the read coverage difference within the FTO^+^ and FTO^–^ samples m2 = (FTO^–^ depth − FTO^+^ depth) / (FTO^–^ depth).
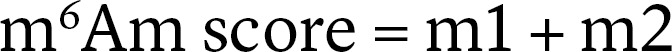


### GO enrichment analysis and motif discovery.

To investigate the relevant biological processes, we performed Gene Ontology (GO) term enrichment analysis using the Database for Annotation, Visualization and Integrated Discovery (DAVID; version 6.8) (https://david.ncifcrf.gov/). We focused on *Homo sapiens* as the selected species and considered biological processes with a *P* value threshold of <0.05. Furthermore, to analyze sequence consensus, we conducted de novo motif analysis with MEME (version 4.12.0) using all peaks (38). The input consisted of 30 nt long peak summit-centered sense sequences.

### Quantification of m^6^A and m^6^Am levels by LC-MS/MS analysis.

One microgram of mRNA was added into lysis buffer containing S1 nuclease (180 U/μL), alkaline phosphatase (30 U/μL), phosphodiesterase (0.002 U/μL), and NH_4_HCO_3_ (1 M) for incubation at 37°C. After the RNA was digested into nucleosides completely, the mixture was extracted with chloroform. The resulting aqueous layer was collected for analysis with LC-ESI-MS/MS, and m^6^A and m^6^Am modification contents were detected by MetWare (http://www.metware.cn/) based on the ABSciex QTRAP 6500 LC-MS/MS platform.

### RNA immunoprecipitation.

Cells were lysed in IP lysis buffer (Beyotime, P0013) supplemented with Protease Inhibitor Cocktail and Anti-RNase. DNase-treated lysate was centrifuged, and 50 μL of the supernatant was stored as input sample. The rest of the supernatant was incubated with 2 μg of specific antibodies or IgG control at 4°C overnight. The RNA-protein-antibody complex was washed 3 times and incubated with Pierce protein A/G magnetic beads (Thermo Fisher Scientific, 88803) for 4 hours at 4°C. The immunoprecipitated RNA was extracted and detected by real-time qPCR.

### GST pull-down.

PCIF1-FLAG plasmid was transfected into SCC1 cells. The lysates of the cells were incubated with GST-CTBP2 or GST control for 2 hours at 4°C, followed by binding to GST beads. After washing 3 times, the bound complex was boiled and analyzed by Western blotting with anti-FLAG (Cell Biological, RM1002) or anti-GST antibodies (Abcam, ab9085).

### Dual luciferase reporter assay.

Cells were cotransfected with pmirGLO-TET2-5′-UTR-WT or pmirGLO-TET2-5′-UTR-MUT reporter plasmids and control vector, PCIF1 gene overexpression plasmid, or PCIF1 catalytic inactive mutant plasmid accordingly. After transfection for 48 hours, cells were harvested and the luciferase activities were measured by the Dual-Luciferase Reporter Assay System (MedChemExpress, HY-K1013).

### Ribo-Seq and data analysis.

Ribosome sequencing (Ribo-Seq) was performed as previously described (22). Briefly, SCC25 cells were incubated with harringtonine (Solarbio), and then cycloheximide (Sigma-Aldrich) was used to block translation. After incubation with RNase I and DNase I at 25°C for 45 minutes, nuclease digestion was stopped with RNase inhibitor (Vazyme). Then, digested ribosome footprints were added to the column and centrifuged at 600*g* for 2 minutes. Ribo-Seq libraries were constructed using NEBNext R Multiple Small RNA Library Prep Set for Illumina R (New England Biolabs). The Ribo-Seq data were analyzed using the RiboTool kit (https://bioinformatics.sc.cn/RiboToolkit). Translation efficiency (TE) ratios between the numbers of ribosome fragments were calculated consistent with the sequence coding for amino acids in protein (CDS) region and mRNA abundance for individual genes. TEs with a 1-fold decrease were considered TE-downregulated genes.

### H&E staining and IHC staining.

All specimens collected were routinely embedded in paraffin, and the tissue slides were deparaffinized and rehydrated. H&E staining was performed with an H&E staining kit (Solarbio, G1120-100). For IHC staining, after incubation with 3% H_2_O_2_, antigen retrieval, and blocking, sections were incubated with primary anti-PCIF1 (Novus, NBP2-13740), anti-CTBP2 (Proteintech, 10346), anti-TET2 (Abcam, ab94580), anti-Ki67 (Novus, NB500-170), and anti-PCK (pan-cytokeratin; Santa Cruz, sc-8018) overnight at 4°C. After washing, secondary antibodies and streptavidin-biotin complex (SABC) were applied with an SABC-POD kit (BOSTER, SA1022). Then the slides were stained with a 3,3′-diaminobenzidine (DAB) kit (BOSTER) and counterstained with hematoxylin. For the IHC analysis, the IHC score was calculated by multiplying the proportion of positively stained cells at each intensity level by their respective intensity score (intensity scores ranging from 0 to 3). The final score ranges from 0 to 300. For Ki67, the percentage of positively stained cells was calculated. The anti-PCK antibody (Santa Cruz Biotechnology, sc-8018) was used for IHC staining of metastatic cells in cervical lymph nodes.

### Immunofluorescence staining.

Cells growing on the glass slide were fixed with 4% paraformaldehyde. Then the slides were stained with primary anti-PCIF1 (Novus, NBP2-13740) and anti-CTBP2 (Proteintech, 10346) antibody overnight in dark. Then the corresponding secondary antibody conjugated with DyLight 488 (Thermo Fisher Scientific, A-11008) and Alexa Fluor 594 (Thermo Fisher Scientific, A-11012) was used to identify corresponding primary antibody. The nuclei were counterstained with DAPI (Solarbio, C0065), and images were captured with an upright fluorescence microscope (Zeiss).

### Quantification of 5hmC and 5mC levels.

Genomic DNA was denatured at 95°C for 5 minutes and added to Hybond N^+^ membrane. Then the membrane was cross-linked twice using the Stratalinker 2400 UV Crosslinker (Stratalinker) with 1,200 μJ for 50 seconds. After blocking with 5% nonfat milk, the membrane was incubated with anti-5mC (Abcam, ab73938) and anti-5hmC (Proteintech, 40900) antibody at 4°C overnight, followed by secondary antibody. The dot blot signal intensity was detected by ECL with Tanon 5200 Multi intelligent imaging system. Then the membrane was stained with 0.02% Methylene Blue (Sigma-Aldrich) and scanned to reveal total input DNA content.

### Statistics.

For bar graphs, all data were presented as the mean ± SD. For statistical comparisons, difference analysis was conducted by means of unpaired parametric 2-tailed Student’s *t* test between 2 groups or χ^2^ for tumor grade analysis. Survival analysis was performed with a Kaplan-Meier curve and log-rank test. The correlations between PCIF1 and CTBP2 expression levels were analyzed with Pearson’s correlation test. *P* less than 0.05 was defined as the threshold for statistical significance. Data management and statistical analyses were performed using GraphPad Prism 8.0 software (GraphPad Software).

### Study approval.

All patients gave informed consent and had not received preoperative radiotherapy or chemotherapy. All animal experiments in this study were approved by the Institutional Animal Care and Use Committee, Sun Yat-sen University.

### Data availability.

The raw sequencing data reported in this paper (including m^6^Am-Seq, Ribo-Seq, and CLIP-Seq) were deposited in the Genome Sequence Archive in the National Genomics Data Center under accession number HRA003566. The raw sequencing data are available for noncommercial purposes under controlled access because of data privacy laws, and access can be obtained by request to the corresponding authors. Values for all data points in graphs are reported in the Supporting Data Values file. This study did not generate new code.

## Author contributions

KL, JC, LP, QC, QY, and DC conceived the study. KL, JC, CZ, MC, SC, CY, ZC, XW, GX, YZ, QL, and DC provided methodology. KL, JC, CZ, MC, SC, RL, JM, QL, and DC analyzed and curated data. KL, JC, CZ, MC, SC, QL, LP, QC, QY, and DC provided investigation and validation. YZ, QL, LP, QC, QY, and DC provided resources. KL, JC, and DC wrote the original draft of the manuscript. KL, JC, QC, and DC reviewed and edited the manuscript. QL, LP, QC, QY, and DC provided supervision and acquired funding.

## Supplementary Material

Supplemental data

Supporting data values

## Version 1 08/29/2023

In-Press Preview

## Version 2 10/16/2023

Electronic publication

## Version 3 07/21/2026

Addition of missing annotations and the standardization of numerical precision in Supporting Data Value file
