# Supplementary material for: The CTBP2-PCIF1 complex regulates m6Am modification of mRNA in head and neck squamous cell carcinoma
Source: J Clin Invest. 2023 Aug 29;133(20):e170173. doi: 10.1172/JCI170173 (PMC10575729; doi:10.1172/JCI170173)
Supplement: Supplemental data [file jci-133-170173-s009.pdf]

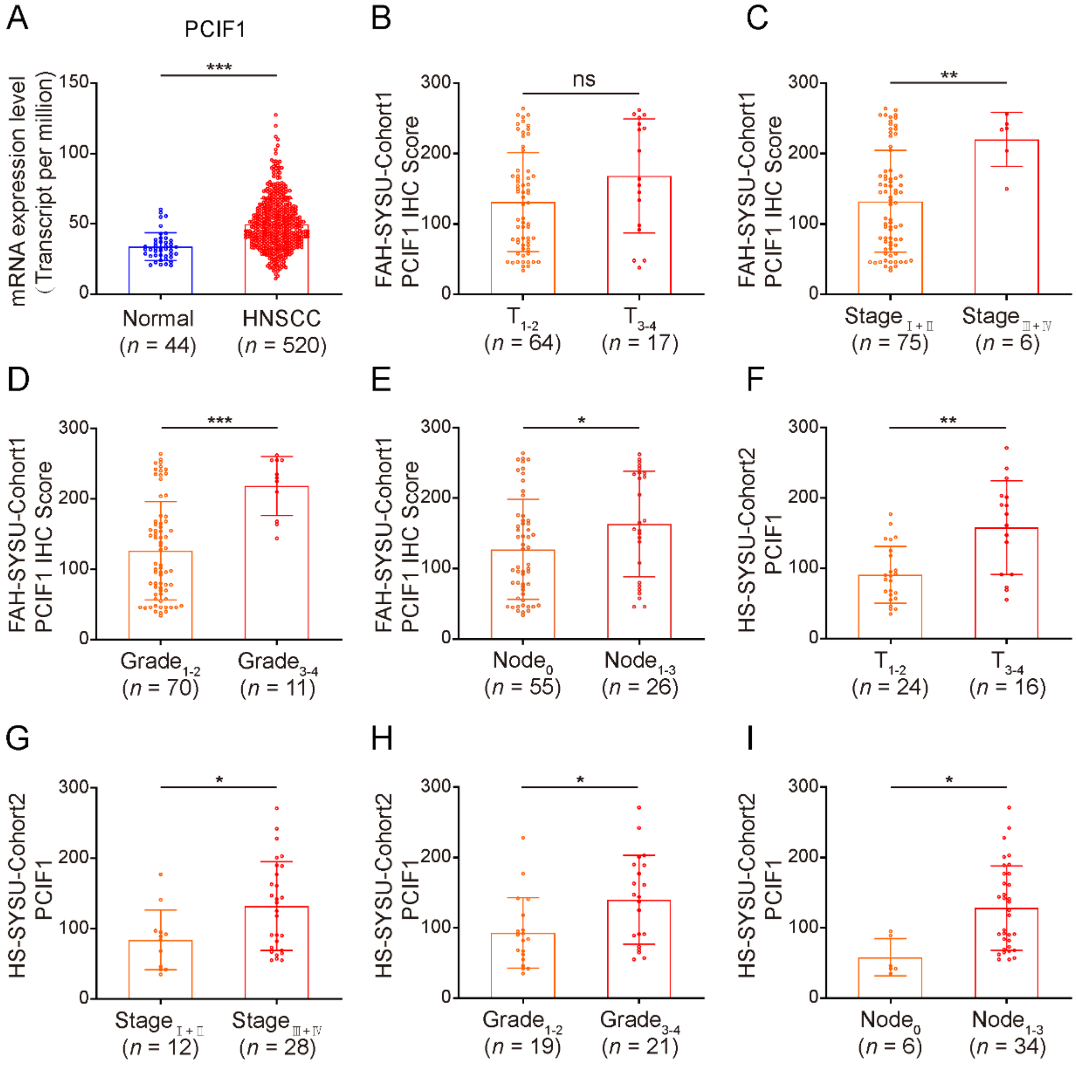

Supplemental Figure 1 Related to Figure 1. PCIF1 expression associated with clinical pathological parameters in HNSCC patients cohorts.

A. PCIF1 mRNA expression in the normal tissues (n = 44) and tumor tissues (n = 520) from The Cancer Genome Atlas (TCGA) head and neck squamous cell carcinoma

profiles. ***$p < 0.001$ by two-tailed unpaired Student's t-test.

B-E. Comparison of PCIF1 staining score by T classification (B), tumor stage (C), tumor grade (D), and lymph node metastasis status (E) of patient from FAH-SYSU-Cohort1 ($n = 81$). $p>0.05$, *$p < 0.05$, **$p < 0.01$, ***$p < 0.001$ by two-tailed unpaired Student's t-test.

F-I. Comparison of PCIF1 staining score by T classification (F), tumor stage (G), tumor grade (H), and lymph node metastasis status (I) of patient from HS-SYSU-Cohort2 ($n = 40$). *$p < 0.05$, **$p < 0.01$ by two-tailed unpaired Student's t-test.

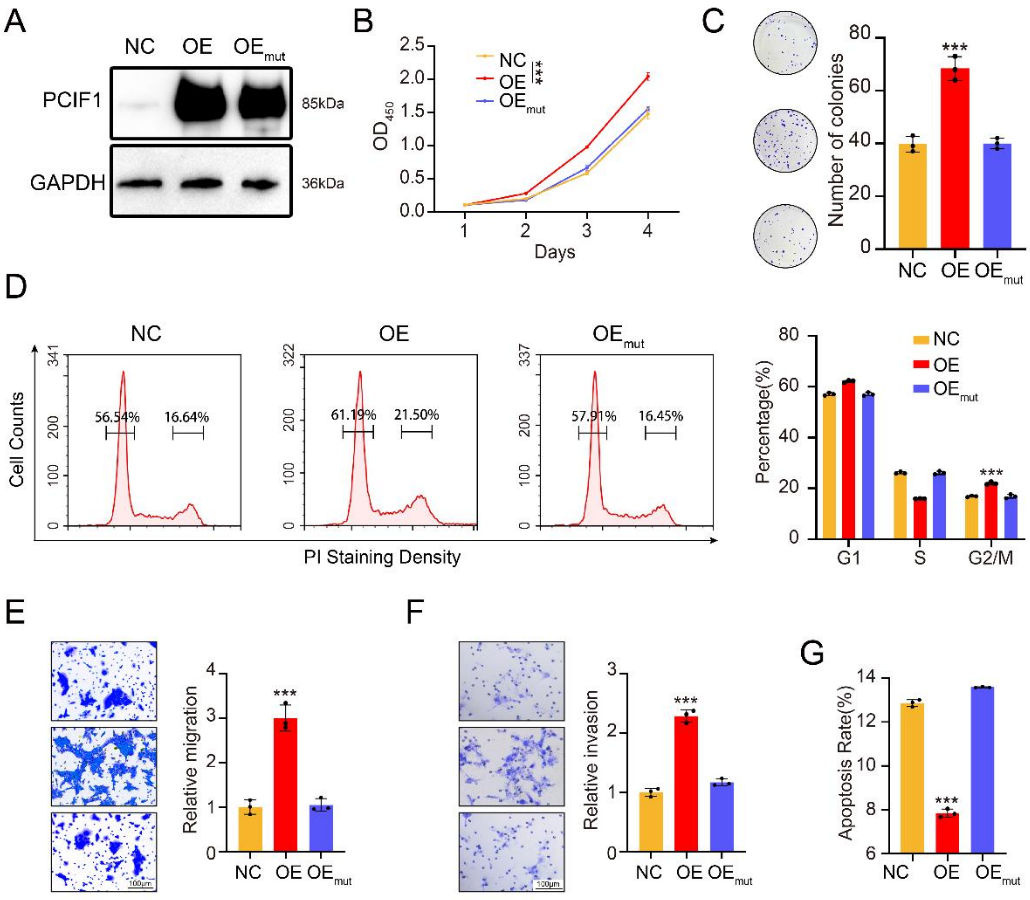

Supplemental Figure 2 Related to Figure 2. PCIF1 played an oncogenic function in m^6Am-dependent manner.

A. Western blotting analyses detecting the PCIF1 expression in SCC1 control cells and transfected with wild-type (OE) or mutant PCIF1 plasmid (OE_{mut}).

B. Cell Counting Kit-8 assay (CCK8) of cell viability in control, OE and OE_{mut} groups. Data are presented as mean ± SD (n = 3). ***p < 0.001 by one-way ANOVA with Tukey's multiple comparison test.

C. The colony formation assay detecting the colony ability of control, OE and OE_{mut} groups. Data are presented as mean ± SD (n = 3). ***p < 0.001 by one-way ANOVA with Tukey's multiple comparison test.

D. The cell cycle progression was detected by flow cytometric analyses in control, OE and OE_{mut} group. Left panels: representative images. Right panel: quantification data. Data are presented as mean ± SD (n = 3). ***p < 0.001 by one-way ANOVA with Tukey's multiple comparison test.

E and F. The cell migration (F) and invasion (G) ability of control, OE and OE_{mut} groups were determined by transwell assay. Data are presented as mean ± SD (n = 3). ***p < 0.001 by one-way ANOVA with Tukey's multiple comparison test.

G. Flow cytometry assay for cell apoptosis in control, OE and OE_{mut} groups. Data are presented as mean ± SD (n = 3). ***p < 0.001 by one-way ANOVA with Tukey's multiple comparison test.

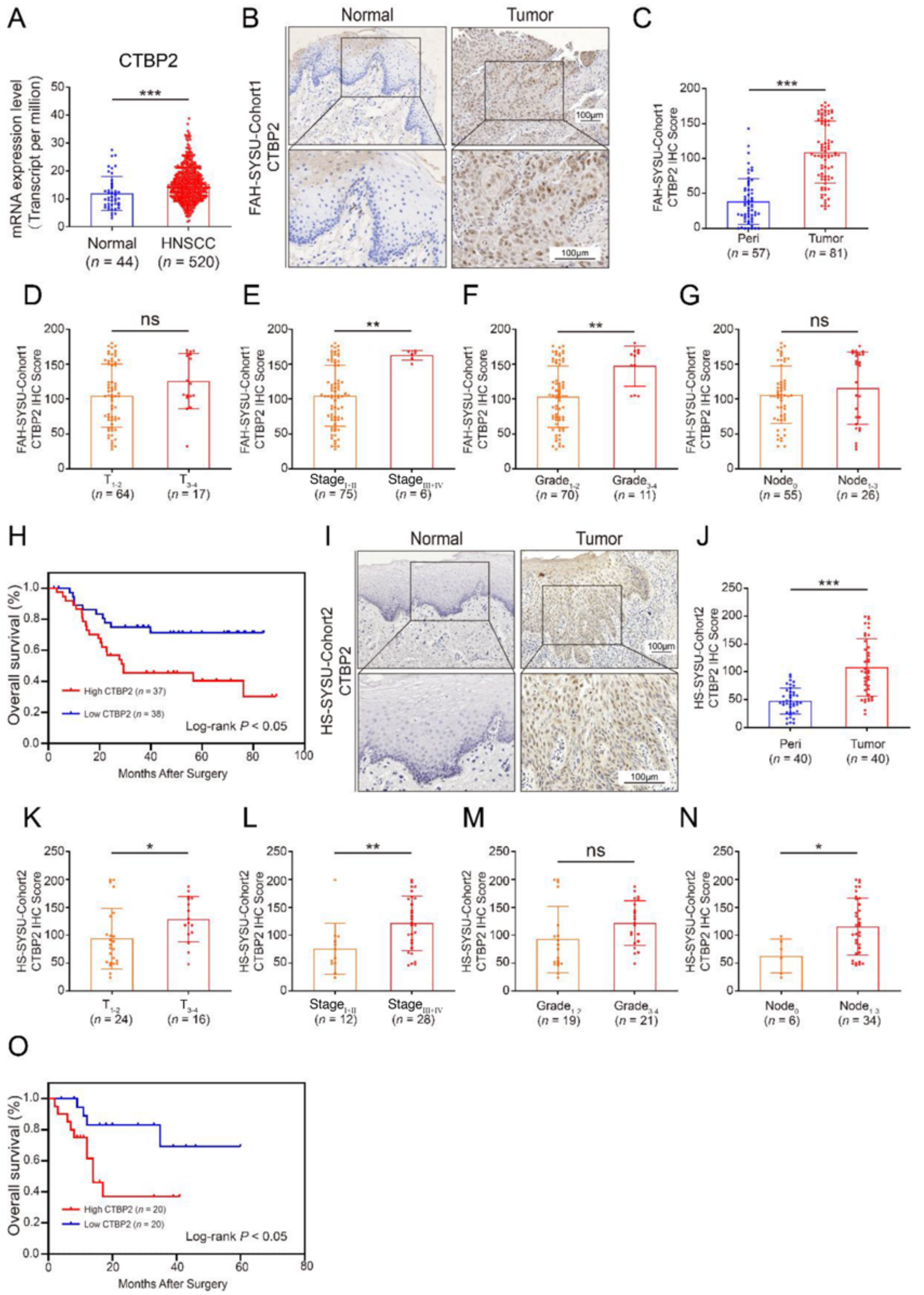

Supplemental Figure 3 Related to Figure 3. Prognostic impact of CTBP2 expression in HNSCC.

A. CTBP2 mRNA expression in the normal tissues (n = 44) and tumor tissues (n = 520) from The Cancer Genome Atlas (TCGA) head and neck squamous cell carcinoma profiles. ***p < 0.001 by two-tailed unpaired Student's t-test.

B. Representative images of CTBP2 staining in tumor and nontumorous tissues from HNSCC patients (FAH-SYSU-Cohort1). Scale bar, 100μm.

C. Quantification of CTBP2 staining score between tumor tissue samples (n = 81) and nontumorous tissue samples (n = 57) from HNSCC patients (FAH-SYSU-Cohort1). ***$p < 0.001$ by two-tailed unpaired Student's t-test.

D-G. Comparison of PCIF1 staining score by T classification (D), tumor stage (E), tumor grade (F), and lymph node metastasis status (G) of patient from FAH-SYSU-Cohort1 (n = 81). $p > 0.05$, **$p < 0.01$ by two-tailed unpaired Student's t-test.

H. Kaplan-Meier curve depicting the overall survival (OS) of patients with HNSCC (FAH-SYSU-Cohort1) stratified by CTBP2 expression levels. P values were calculated by log-rank test.

I. Representative images of CTBP2 staining in tumor and nontumorous tissues from HNSCC patients (HS-SYSU-Cohort2). Scale bar, 100μm.

J. Quantification of CTBP2 staining score between tumor tissue samples (n = 40) and nontumorous tissue samples (n = 40) from HNSCC patients (HS-SYSU-Cohort2). ***p < 0.001 by two-tailed unpaired Student's t-test.

K-N. Comparison of PCIF1 staining score by T classification (K), tumor stage (L), tumor grade (M), and lymph node metastasis status (N) of patient from HS-SYSU-Cohort2 (n = 40). $p > 0.05$, *$p < 0.05$, **$p < 0.01$ by two-tailed unpaired Student's t-test.

O. Kaplan-Meier curve depicting the overall survival (OS) of patients with HNSCC (HS-SYSU-Cohort2) stratified by CTBP2 expression levels. *P* values were calculated by log-rank test.

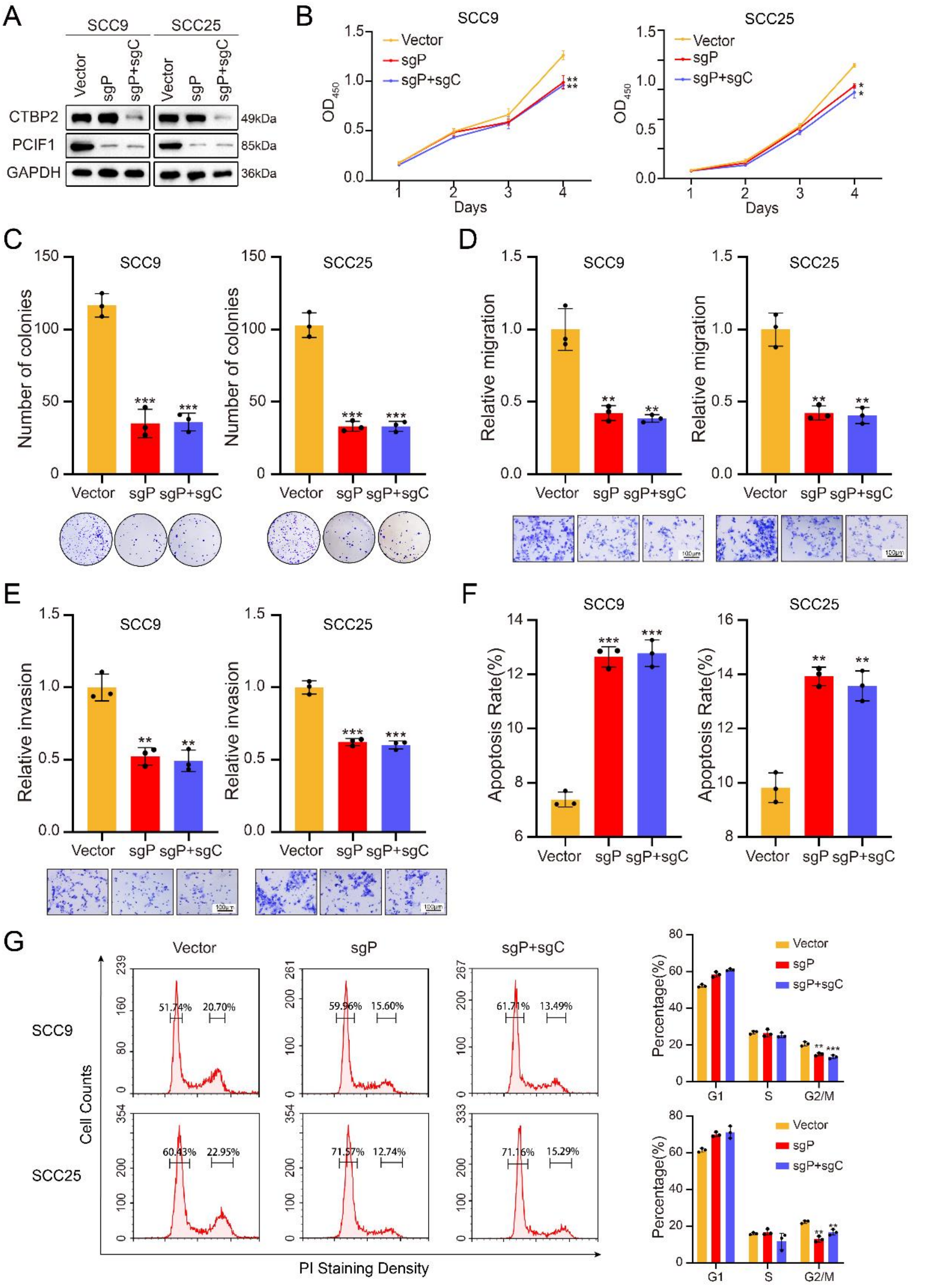

Supplemental Figure 4 Related to Figure 3. The dual knockdown of PCIF1 and CTBP2 did not synergistically worsen the phenotype resulting from PCIF1 knockdown.

A. PCIF1 and CTBP2 protein expression level in HNSCC cell lines with sole PCIF1 knockout (sgP) or dual knockout of PCIF1 and CTBP2 (sgP + sgC).

B-F. The proliferation ability (B), migration (C), invasion (D), cell apoptosis (E), and cell cycle (F) were detected in HNSCC cell lines with sole PCIF1 knockout or dual

knockout of PCIF1 and CTBP2. Data are presented as mean ± SD (n = 3). *$p < 0.05$, **$p < 0.01$ by one-way analysis of variance, Dunnett's test (B). *$p < 0.05$, **$p < 0.01$, ***$p < 0.001$ by one-way ANOVA with Tukey's multiple comparison test (C-G).

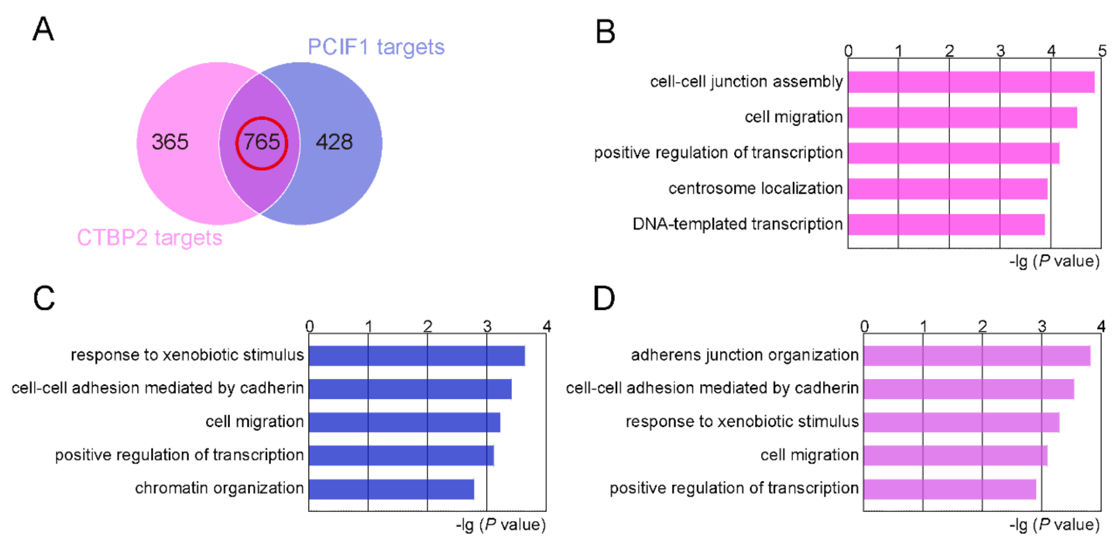

Supplemental Figure 5 Related to Figure 3. PCIF1 and CTBP2 CLIP-seq in SCC25 cells.

A. Venn diagrams shows the intersections of genes in CTBP2 CLIP-seq targets (CTBP2 targets) and PCIF1 CLIP-seq targets (PCIF1 targets).

B-D. Bar plots showing the top 5 GO terms of CTBP2 CLIP-seq targets genes (B), PCIF1 CLIP-seq targets genes (C), and the overlapped genes (D).

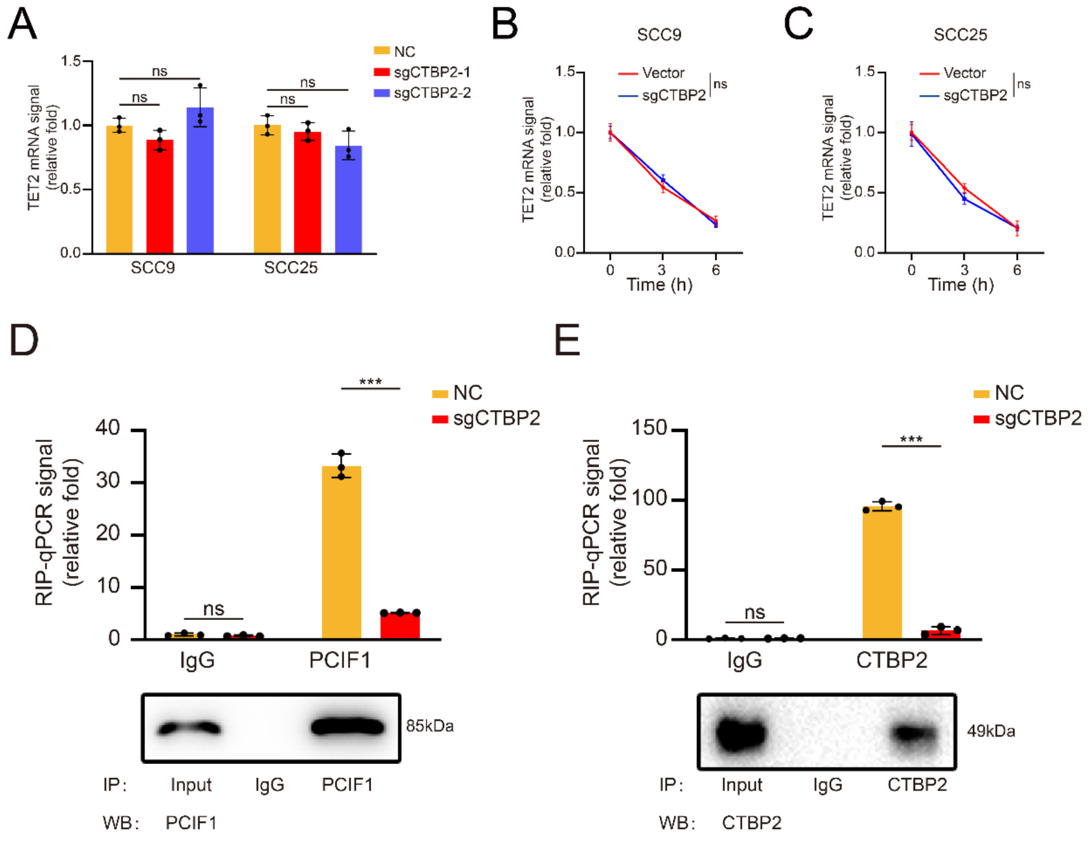

Supplemental Figure 6 Related to Figure 5. TET2 transcriptional activity is unaffected after CTBP2 knockout.

A. TET2 mRNA expression was determined by qRT-PCR in control and CTBP2 knockout cells. Data are presented as mean ± SD (n = 3). $p > 0.05$ by one-way analysis

of variance, Dunnett's test.

B. qRT-PCR analysis of TET2 mRNA levels at the indicated times in SCC9 control and CTBP2 knockout cells after actinomycin D treatment. Data are presented as mean ± SD (n = 3). $p > 0.05$ by two-tailed unpaired Student's t-test.

C. qRT-PCR analysis of TET2 mRNA levels at the indicated times in SCC25 control and CTBP2 knockout cells after actinomycin D treatment. Data are presented as mean ± SD (n = 3). $p > 0.05$ by two-tailed unpaired Student's t-test.

D. RIP-qPCR analysis of TET2 mRNA retrieved by anti-PCIF1 antibody in control and CTBP2 knockout cells. Data are presented as mean ± SD (n = 3). $p > 0.05$, ***$p < 0.001$ were obtained from two-tailed unpaired Student's t-test.

E. RIP-qPCR analysis of TET2 mRNA retrieved by anti-CTBP2 antibody in control and CTBP2 knockout cells. Data are presented as mean ± SD (n = 3). $p > 0.05$, ***$p < 0.001$ were obtained from two-tailed unpaired Student's t-test.

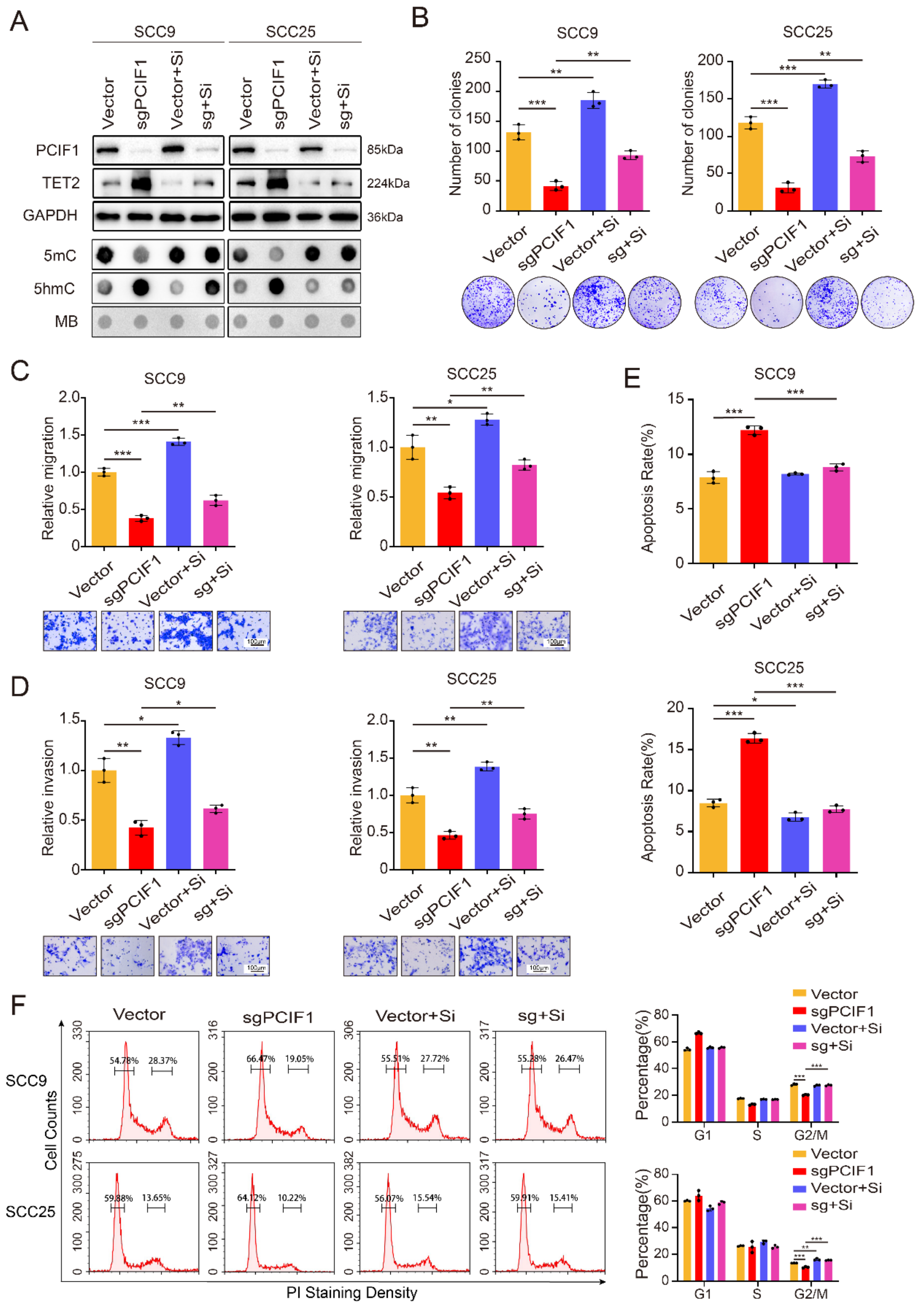

Supplemental Figure 7 Related to Figure 5. Identification of TET2 as a functional target in HNSCC cell lines.

A. PCIF1 and TET2 protein expression level (upper) and DNA 5mC and 5hmC modification levels (lower) in HNSCC cell lines with or without stable PCIF1 knockout

was transfected with non-targeting siRNA or siRNA targeting TET2.

B-F. The proliferation ability (B), migration (C), invasion (D), cell apoptosis (E), and cell cycle (F) were detected in HNSCC cell lines with or without stable PCIF1 knockout was transfected with non-targeting siRNA or siRNA targeting TET2. Data are presented as mean ± SD (n = 3). *$p < 0.05$, **$p < 0.01$, ***$p < 0.001$ by one-way ANOVA with Tukey's multiple comparison test.

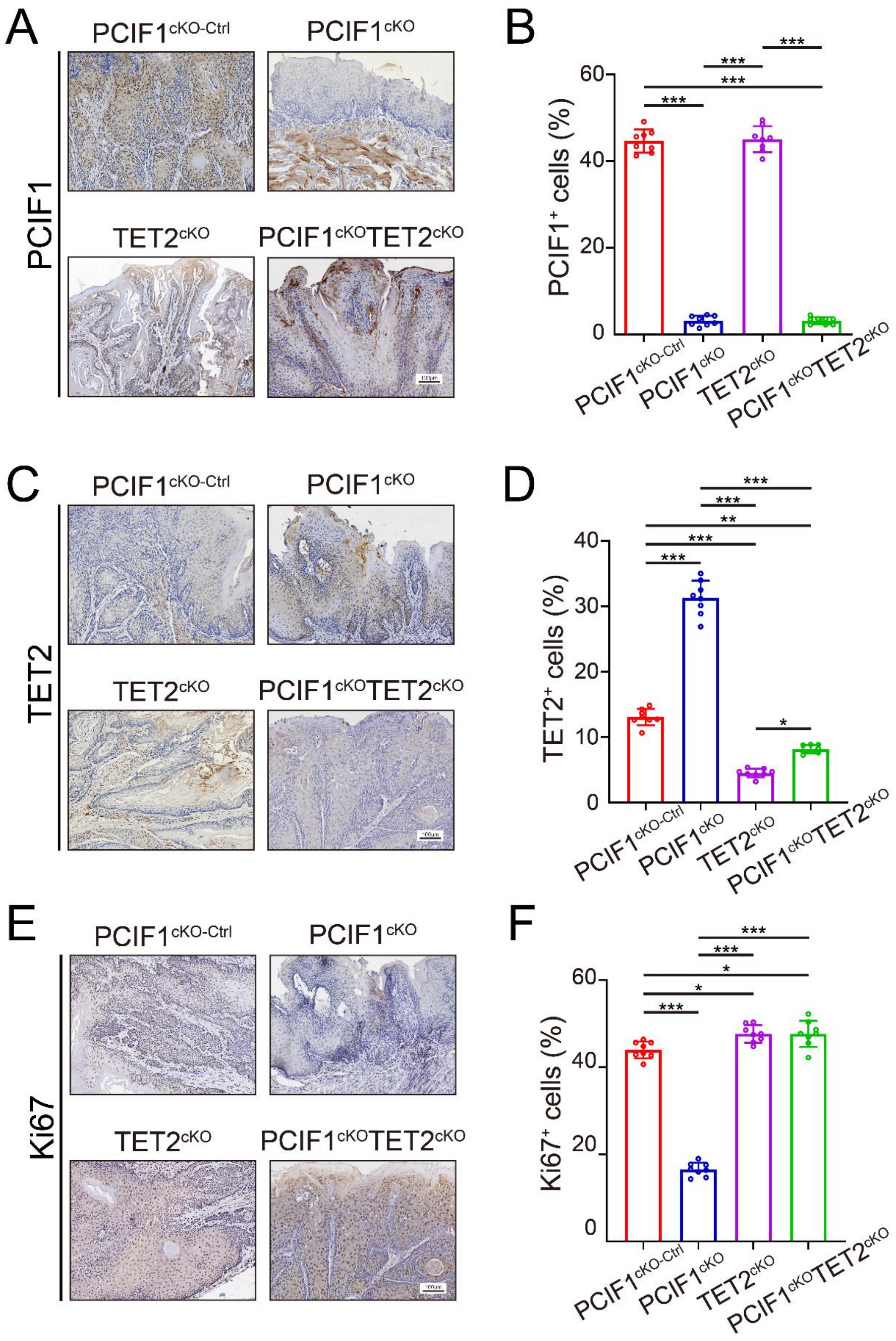

Supplemental Figure 8 Related to Figure 6. Effects of *PCIF1* knockout on TET2 expression and cell proliferation.

A. Representative PCIF1 staining of HNSCC in the indicated groups. Scale bar, 100μm.

B. Quantification of PCIF1^{+} cells in the indicated groups. Data are presented as mean

± SD (n = 8). ***p < 0.001 by one-way ANOVA with Tukey's multiple comparison test.

C. Representative TET2 staining of HNSCC in the indicated groups. Scale bar, 100μm.

D. Quantification of $TET2^{+}$ cells in the indicated groups. Data are presented as mean ± SD (n = 8). *p < 0.05, **p<0.01, ***p < 0.001 by one-way ANOVA with Tukey's multiple comparison test.

E. Representative Ki67 staining of HNSCC in the indicated groups. Scale bar, 100μm.

F. Quantification of $Ki67^{+}$ cells in the indicated groups. Data are presented as mean ± SD (n = 8). *p < 0.05, ***p < 0.001 by one-way ANOVA with Tukey's multiple comparison test.

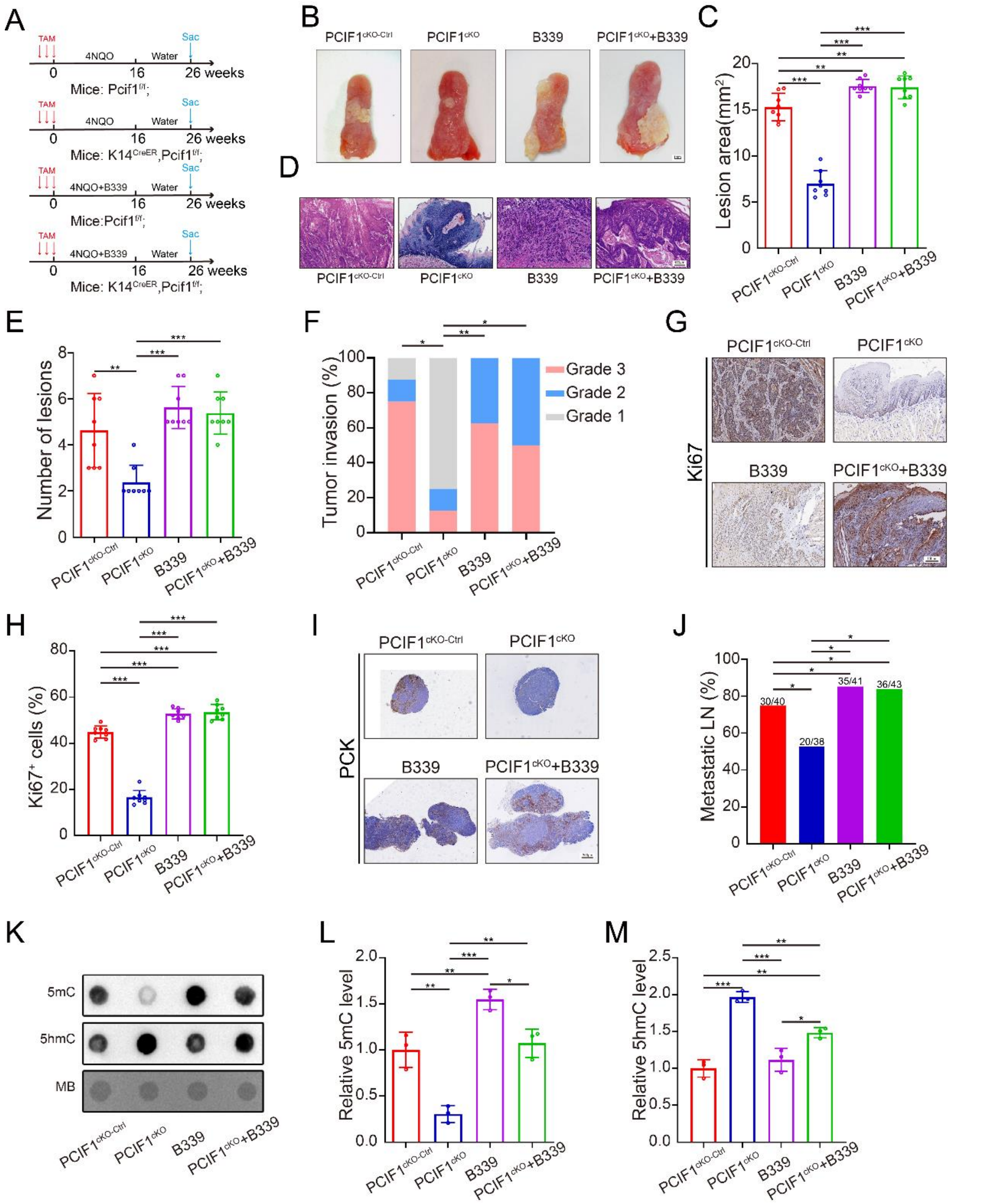

Supplemental Figure 9 Related to Figure 6. Effects of TET2 inhibitor on tumor growth and metastasis in *Pcif1* knockout mice.

A. The experimental design showing the B339 treatment schedule for the carcinogen-induced HNSCC mouse model.

B. Representative image of tongue visible lesions in the different treatment groups. Scale bar, 1mm.

C. Quantification of HNSCC lesion area in the different treatment groups. Data are presented as mean ± SD (n = 8). $^{**}p < 0.01$, $^{***}p < 0.001$ by one-way ANOVA with

Tukey's multiple comparison test.

D. Representative H&E staining of HNSCC in the different treatment groups. Scale bar, 100μm.

E. Quantification of HNSCC number of lesions in the different treatment groups. Data are presented as mean ± SD (n = 8). $^{**}p < 0.01$, $^{***}p < 0.001$ by one-way ANOVA with Tukey's multiple comparison test.

F. Quantification of HNSCC tumor grade in the different treatment groups. $^{*}p < 0.05$, $^{**}p < 0.01$ by Pearson chi-square test.

G. Representative Ki67 staining of HNSCC in the different treatment groups. Scale bar, 100μm.

H. Quantification of $Ki67^+$ cells in the different treatment groups. Data are presented as mean ± SD (n = 8). $^{***}p < 0.001$ by one-way ANOVA with Tukey's multiple comparison test.

I. Representative PCK staining of metastatic lymph node in the different treatment groups. Scale bar, 300μm.

J. Quantification of metastatic lymph node percentage in the different treatment groups. $^{*}p < 0.05$ by Pearson chi-square test.

K-M. Representative dot-blot image (K) and the quantitative analysis of DNA 5mC (L) and 5hmC (M) modification levels in the indicated groups. Data are presented as mean ± SD (n = 3). $^{*}p < 0.05$, $^{**}p < 0.01$, $^{***}p < 0.001$ by one-way ANOVA with Tukey's multiple comparison test.

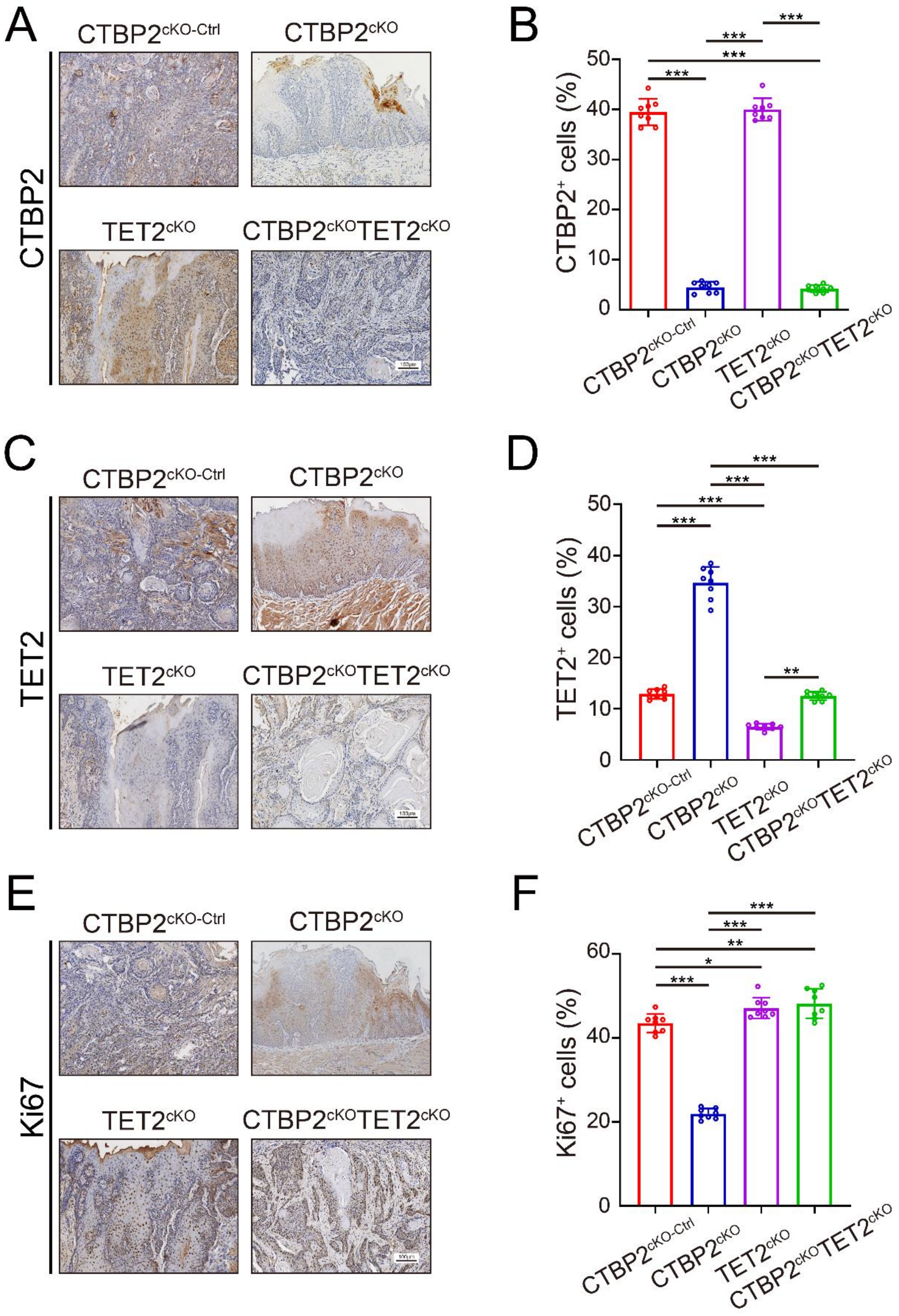

Supplemental Figure 10 Related to Figure 7. Effects of CTBP2 knockout on TET2 expression and cell proliferation.

A. Representative CTBP2 staining of HNSCC in the indicated groups. Scale bar, 100μm.

B. Quantification of $CTBP2^{+}$ cells in the indicated groups. Data are presented as mean

± SD (n = 8). ***$p < 0.001$ by one-way ANOVA with Tukey's multiple comparison test.

C. Representative TET2 staining of HNSCC in the indicated groups. Scale bar, 100μm.

D. Quantification of TET2$^+$ cells in the indicated groups. Data are presented as mean ± SD (n = 8). **$p < 0.01$, ***$p < 0.001$ by one-way ANOVA with Tukey's multiple comparison test.

E. Representative Ki67 staining of HNSCC in the indicated groups. Scale bar, 100μm.

F. Quantification of Ki67$^+$ cells in the indicated groups. Data are presented as mean ± SD (n = 8). *$p < 0.05$, **$p < 0.01$, ***$p < 0.001$ by one-way ANOVA with Tukey's multiple comparison test.

Supplemental Table 1: The demographic, pathologic, and clinical information of FAH-SYSU cohort.

ID	gender	age	Tumor	grade	stage	lymph node	time	status
1	Male	62	2	1	2	0	56.6	0
2	Male	45	1	1	1	0	30	0
3	Female	63	3	1	2	0	39.8	1
4	Male	53	1	1	1	0	76.8	0
5	Male	29	2	1	2	2	76.2	1
6	Female	46	3	1	2	2	9.57	1
7	Male	56	1	1	1	0	13.23	1
8	Male	56	1	1	1	0	-	-
9	Male	50	4	1	3	0	3.27	1
10	Male	51	4	1	3	2	38.3	0
11	Male	61	1	1	1	0	73.6	0
12	Male	59	1	2	1	0	10.83	1
13	Male	62	1	2	1	0	14.9	1
14	Male	83	1	2	1	0	35	0
15	Male	64	1	1	1	0	24.23	0
16	Male	85	1	2	1	0	37.2	0
17	Male	66	1	1	1	0	22.9	1
18	Male	68	1	1	1	0	83.8	0
19	Female	80	1	2	1	0	34.6	0
20	Male	72	1	1	1	0	10	1
21	Male	41	2	2	1	0	2.2	0
22	Male	35	2	1	1	0	27.67	1
23	Male	47	2	2	1	0	87.4	0
24	Male	52	2	1	1	0	89	0
25	Male	60	2	1	1	0	50.9	0
26	Male	61	2	1	1	0	74.3	0
27	Female	23	2	1	1	0	65.7	0
28	Female	59	2	1	1	0	48.8	0
29	Female	65	2	1	1	0	52.4	0
30	Male	49	2	2	1	0	65.6	0
31	Male	62	2	2	1	0	37	0
32	Male	65	2	2	1	0	29.43	1
33	Female	61	1	1	1	1	14.73	1
34	Male	46	3	1	2	0	76.4	0
35	Male	68	2	2	1	0	46.7	0
36	Male	63	3	1	2	0	19.63	1
37	Male	68	3	1	2	0	55.7	0
38	Female	50	3	1	2	0	21.27	1
39	Male	47	1	2	1	0	69.9	0
40	Female	63	2	2	1	0	22	1

41	Female	85	2	2	1	0	-	-
42	Male	42	3	2	2	0	28.63	1
43	Male	42	3	2	2	0	22.67	1
44	Female	59	1	2	2	2	13.5	1
45	Female	70	1	2	2	2	15.97	1
46	Male	62	2	2	2	2	79.7	0
47	Male	66	2	2	2	2	8.4	1
48	Male	70	2	2	2	2	49	0
49	Male	41	4	2	3	0	47.7	0
50	Female	82	4	2	3	1	4.03	0
51	Male	50	1	2	1	0	20.47	1
52	Male	67	1	2	1	0	70.6	0
53	Female	53	2	2	3	3	13.5	1
54	Male	88	1	2	1	0	84.1	0
55	Male	59	4	2	3	3	9.83	1
56	Female	63	1	2	1	0	59.9	0
57	Female	63	1	2	1	0	48.9	0
58	Male	32	2	2	1	0	50.3	1
59	Male	44	1	3	1	0	6.7	1
60	Male	73	1	3	1	0	18.77	1
61	Male	66	2	2	1	0	76.9	0
62	Female	54	1	3	1	0	36.9	0
63	Male	71	2	2	1	0	80.3	0
64	Male	48	1	2	1	1	60.2	0
65	Female	62	1	3	1	0	29.37	1
66	Female	51	1	2	1	1	24.53	1
67	Female	78	1	2	1	1	10	1
68	Male	29	2	3	1	1	38.4	0
69	Male	66	3	3	2	0	5.63	1
70	Male	37	2	2	1	1	71.2	0
71	Male	62	2	2	1	1	-	-
72	Male	53	1	3	2	2	-	-
73	Male	60	2	3	2	2	40	0
74	Male	63	2	2	1	1	13.03	1
75	Female	69	2	2	1	1	87.4	0
76	Male	82	3	2	2	0	-	-
77	Male	63	2	3	2	2	-	-
78	Female	47	3	2	2	0	68.7	0
79	Female	46	2	2	2	2	41.2	0
80	Female	64	2	3	2	2	39.1	0
81	Male	70	3	3	2	2	20.9	1

Supplemental Table 2: The demographic, pathologic, and clinical information of HS-SYSU cohort.

ID	gender	age	Tumor	grade	stage	lymph node	time	status
20150780	Female	62	3	3	3	1	6	1
20150732	Female	54	4	3	4	1	9	0
20150611	Male	41	3	3	3	2	2	1
20150655	Female	53	1	1	1	0	8	0
20150631	Female	60	2	1	2	1	4	0
20150451	Male	21	3	1	3	1	9	1
20150412	Male	74	2	2	3	2	18	0
20150453	Male	74	2	3	3	2	10	0
20150382	Female	75	3	3	3	2	7	1
20150283	Female	47	1	1	1	0	33	0
20150181	Male	64	4	3	4	1	39	0
20141575	Female	63	1	1	3	2	14	1
20141423	Male	59	2	1	3	2	12	0
20141404	Male	68	2	3	3	2	39	0
20141247	Male	60	2	3	3	2	16	0
20141125	Male	49	2	3	3	2	43	0
20140915	Male	51	2	1	2	1	12	0
20140753	Female	68	2	1	2	1	12	0
20140721	Male	52	3	3	3	2	3	1
20140620	Male	64	3	3	3	2	41	0
20140507	Male	68	3	3	3	2	17	0
20131381	Male	55	2	2	3	2	17	1
20130760	Female	32	2	3	3	2	11	1
20130738	Male	43	4	3	4	2	11	0
20130538	Male	59	4	3	4	2	8	1
20130399	Male	40	2	1	3	2	12	1
20130339	Male	32	2	1	2	1	35	1
20120695	Male	64	2	3	3	2	12	1
20110927	Male	56	2	1	2	1	28	0
20110731	Male	66	3	1	3	1	33	0
20150656	Female	55	1	1	3	2	16	0
20150282	Male	53	3	3	3	1	14	1
20111017	Male	49	2	1	2	1	46	0
20151029	Male	62	1	1	1	0	11	0
20141592	Male	60	3	3	3	2	20	0
20141389	Male	57	4	3	4	3	8	0
20141166	Male	55	1	1	1	0	18	0
20151032	Male	42	2	3	2	0	60	0
20150630	Female	57	2	1	2	0	60	0
20150522	Female	35	3	3	3	1	12	1

Supplemental Table 3: MS-iTRAQ analysis results.

Protein_ID	Protein_Qscore	PepIsUnique	Abundance
sp|P56545|CTBP2_HUMAN	17.86634503	1;1;1;1;1;0	12967607.68
sp|Q14011|CIRBP_HUMAN	6.890291984	1;1	379747.0682
sp|P27695|APEX1_HUMAN	17.91180731	1;1;1;1;1	3778713.687
sp|P67809|YBOX1_HUMAN	15.22498509	1;1;1;1	2262219.914
sp|P07948|LYN_HUMAN	8.866021122	1;1;1	2089401.448
sp|P33176|KINH_HUMAN	11.10673706	1;1;1	229891.1317
sp|P45974|UBP5_HUMAN	10.33543798	1;1;1	502698.3195
sp|P23368|MAOM_HUMAN	10.38090025	1;1;1	1833122.697
sp|Q8WZ19|BACD1_HUMAN	8.10862357	1;1;1	3194754.538
sp|Q9H4Z3|CAPAM_HUMAN	10.73844642	1;1;1	1328905.234
sp|Q9NVI7|ATD3A_HUMAN	9.697053732	1;1;1	923787.4745
sp|Q15758|AAAT_HUMAN	5.760216524	1;1	421909.4197
sp|Q5SRE5|NU188_HUMAN	6.398600768	1;1	1148988.377
sp|P00441|SODC_HUMAN	7.931684669	1;1	1353921.824
sp|Q8TAQ2|SMRC2_HUMAN	6.25190774	1;1	148498.5736
sp|P42126|ECI1_HUMAN	7.239305003	1;1	1796565.063
sp|O00154|BACH_HUMAN	5.61752421	1;1	1683605.095
sp|P50579|MAP2_HUMAN	5.894361572	1;1	523174.5101
sp|Q13724|MOGS_HUMAN	6.571218094	1;1	78383.74169
sp|Q9H307|PININ_HUMAN	5.253760558	1;1	365813.6695
sp|Q9BZF1|OSBL8_HUMAN	5.094780556	1;1	135865.7458
sp|O43169|CYB5B_HUMAN	6.024484338	1;1	16630637.06
sp|Q13057|COASY_HUMAN	6.25190774	1;1	26947.86011
sp|P04114|APOB_HUMAN	4.846351637	1;1	611653.2675
sp|P12236|ADT3_HUMAN	4.486538677	1;0;0;0	98910.26589
sp|P47755|CAZA2_HUMAN	3.04436838	1;0	0
sp|P52789|HXK2_HUMAN	2.068699384	0;1;0	551969.0673
sp|Q9UKA9|PTBP2_HUMAN	2.953454776	0;1	487717.8596
sp|P61981|1433G_HUMAN	7.931684669	0;0;1;0;0;1	4565818.95
sp|P36873|PP1G_HUMAN	2.07558123	0;0;1	302466.8677
sp|Q13838|DX39B_HUMAN	2.953454776	0;0;0;0;0;0;0;1	50594.79353
sp|P48668|K2C6C_HUMAN	3.175052389	0;0;0;0;0;0;0;1;0;0	363046.5312
sp|Q13098|CSN1_HUMAN	3.445145992	1	1605362.882
sp|Q9H299|SH3L3_HUMAN	4.486538677	1	137774.4351
sp|Q15166|PON3_HUMAN	3.04436838	1	445861.0693
sp|Q96P48|ARAP1_HUMAN	2.752766326	1	2240570.591
sp|O94925|GLSK_HUMAN	3.445145992	1	16461.60924
sp|O43148|MCES_HUMAN	3.445145992	1	150083.8525
sp|Q10471|GALT2_HUMAN	2.141325781	1	0
sp|Q8TC12|RDH11_HUMAN	3.445145992	1	43406.96455
sp|Q9BVJ6|UT14A_HUMAN	3.445145992	1	0
sp|Q71RC2|LARP4_HUMAN	3.445145992	1	0

sp|O75964|ATP5L_HUMAN	3.445145992	1	227381.9831
sp|P24534|EF1B_HUMAN	3.445145992	1	1826131.028
sp|Q9UNL2|SSRG_HUMAN	3.445145992	1	1282396.013
sp|P16298|PP2BB_HUMAN	3.445145992	1	113746.7435
sp|P48729|KC1A_HUMAN	3.445145992	1	278827.9722
sp|Q9Y3B4|SF3B6_HUMAN	4.486538677	1	15454.5462
sp|Q9HD45|TM9S3_HUMAN	3.445145992	1	65643.29429
sp|Q14690|RRP5_HUMAN	4.486538677	1	526524.6327
sp|P51649|SSDH_HUMAN	2.547807344	1	498139.8743
sp|P41240|CSK_HUMAN	3.445145992	1	29553.84945
sp|Q8WX92|NELFB_HUMAN	3.445145992	1	0
sp|O95754|SEM4F_HUMAN	2.198412574	1	504079.5662
sp|Q9NPD3|EXOS4_HUMAN	3.04436838	1	315197.1307
sp|Q96C86|DCPS_HUMAN	2.752766326	1	266981.2115
sp|Q96G03|PGM2_HUMAN	2.305521309	1	3064058.832
sp|P48637|GSHB_HUMAN	2.953454776	1	61628.63901
sp|Q99436|PSB7_HUMAN	2.953454776	1	173654.3687
sp|Q15582|BGH3_HUMAN	2.806761748	1	170539.6011
sp|Q8TC07|TBC15_HUMAN	2.467420355	1	64515.74859
sp|Q8NCW5|NNRE_HUMAN	2.256252121	1	3883.422477
sp|P24752|THIL_HUMAN	3.445145992	1	112000.9648
sp|Q9H9S4|CB39L_HUMAN	2.953454776	1	5679.497968
sp|P35080|PROF2_HUMAN	4.486538677	1	1042761.605
sp|Q16666|IF16_HUMAN	2.579338346	1	185629.7297
sp|Q12765|SCRN1_HUMAN	3.445145992	1	207724.0141
sp|Q8IXB1|DJC10_HUMAN	3.445145992	1	0
sp|Q9NS69|TOM22_HUMAN	3.445145992	1	61752.56106
sp|P05386|RLA1_HUMAN	4.486538677	1	346115.1222
sp|P69905|HBA_HUMAN	3.445145992	1	55431.68174
sp|Q9H0A0|NAT10_HUMAN	2.172378218	1	286442.6441
sp|Q9NZP5|O5AC2_HUMAN	2.579338346	1	75440.86844
sp|O75976|CBPD_HUMAN	3.04436838	1	3992392.55
sp|P62308|RUXG_HUMAN	4.486538677	1	4053927.384
sp|Q86YZ3|HORN_HUMAN	3.445145992	1	0
sp|P35251|RFC1_HUMAN	2.953454776	1	0
sp|Q9H1E3|NUCKS_HUMAN	2.655999298	1	4626423.663
sp|Q9Y606|TRUA_HUMAN	2.579338346	1	1023803.297
sp|Q5TA45|INT11_HUMAN	3.445145992	1	13444.26123
sp|P45973|CBX5_HUMAN	2.07558123	1	225493.8538
sp|P41567|EIF1_HUMAN	3.445145992	1	1892859.831
sp|Q9HCY8|S10AE_HUMAN	2.467420355	1	26954.99102
sp|P48651|PTSS1_HUMAN	3.445145992	1	0
sp|P13807|GYS1_HUMAN	2.953454776	1	490729.0189
sp|P54840|GYS2_HUMAN	2.22565399	1	0

sp|Q96EK6|GNA1_HUMAN	2.953454776	1	0
sp|O00217|NDUS8_HUMAN	2.184592397	1	13869.36359
sp|O60502|OGA_HUMAN	2.579338346	1	0
sp|Q96HU8|DIRA2_HUMAN	2.068699384	1	17430115.03
sp|O60678|ANM3_HUMAN	2.467420355	1	0
sp|P04196|HRG_HUMAN	2.305521309	1	0
sp|P14324|FPPS_HUMAN	2.485411303	1	4278643.519
sp|P56134|ATPK_HUMAN	3.445145992	1	894629.9894
sp|P82673|RT35_HUMAN	3.445145992	1	0
sp|Q13243|SRSF5_HUMAN	3.445145992	1	650589.3265
sp|P49406|RM19_HUMAN	2.122609745	1	351257.301
sp|Q8IXI1|MIRO2_HUMAN	2.953454776	1	107631.7671
sp|Q15428|SF3A2_HUMAN	2.953454776	1	123222.2421
sp|Q9BXB4|OSB11_HUMAN	3.445145992	1	0
sp|P53985|MOT1_HUMAN	3.445145992	1	437995.9409
sp|P63220|RS21_HUMAN	3.445145992	1	483649.9964
sp|Q9NXG2|THUM1_HUMAN	4.486538677	1	0

Supplemental Table 4: Primer and oligonucleotides sequences.

Oligonucleotides	sequence
h-TET2-Forward Primer	5’- GATAGAACCAACCATGTTGAGGG-3’
h-TET2-Reverse Primer	5’-TGGAGCTTTGTAGCCAGAGGT-3’
h-GAPDH-Forward Primer	5’-AGATCCCTCCAAAATCAAGTGG-3’
h-GAPDH- Reverse Primer	5’- GGCAGAGATGATGACCCTTTT-3’
si-TET2	5’-CCAUCACAAUUGCUUCUUU-3’
sg-PCIF1-1	5’- CACCTAGCGGTAAAGGAGCCACTG-3’
sg-PCIF1-2	5’- CACCCGGTTGAAAGACTCCCGTGG-3’
sg-CTBP2-1	5’- CACCCGGTTGAAAGACTCCCGTGG-3
sg-CTBP2-2	5’- CGTCGACTGCGCGTCACAGA-3
